# Preparation, characterization and antioxidant and anticancerous potential of Quercetin loaded β-glucan particles derived from mushroom and yeast

**DOI:** 10.1038/s41598-024-66824-1

**Published:** 2024-07-11

**Authors:** Rashmi Trivedi, Tarun Kumar Upadhyay

**Affiliations:** Department of Biotechnology, Parul Institute of Applied Sciences and Research and Development Cell, Parul University, Vadodara, Gujarat 391760 India

**Keywords:** β-glucan, Cell migration, Hemolysis, Drug release, Quercetin, DNA fragmentation, Apoptosis, Prostate cancer, Biological techniques, Biotechnology, Drug discovery, Molecular biology

## Abstract

β-glucans are polysaccharides found in the cell walls of various fungi, bacteria and cereals. β-glucan have been found to show various kinds of anti-inflammatory, antimicrobial, antidiabetic antioxidant and anticancerous activities. In the present study, we have isolated β-glucan from the baker’s yeast *Saccharomyces cerevisiae* and white button mushroom *Agaricus bisporus* and tested their antioxidant potential and anticancerous activity against prostate cancer cell line PC3. Particles were characterized with zeta sizer and further with FTIR that confirmed that the isolated particles are β-glucan and alginate sealing made slow and sustained release of the Quercetin from the β-glucan particles. Morphological analysis of the hollow and Quercetin loaded β-glucan was performed with the SEM analysis and stability was analyzed with TGA and DSC analysis that showed the higher stability of the alginate sealed particles. Assessments of the antioxidant potential showed that Quercetin loaded particles were having higher antioxidant activity than hollow β-glucan particles. Cell viability of the PC3 cells was examined with MTT assay and it was found that Quercetin loaded alginate sealed *Agaricus bisporus* derived β-glucan particles were having lowest IC50. Further ROS generation was found to increase in a dose dependent manner. Apoptosis detection was carried out with Propidium iodide and AO/EtBr staining dye which showed significant death in the cells treated with higher concentration of the particles. Study showed that particles derived from both of the sources were having efficient anticancer activity and showing a dose dependent increase in cell death in PC3 cells upon treatment.

## Introduction

Prostate cancer is characterized by the abnormal growth in prostate gland which is a reproductive organ located in the pelvic canal and is the third leading cause of cancer-related death worldwide. It is the second most common cancer in men and the fourth most common worldwide. There are various factors such as age, family history, smoking, and genetics, which can be considered as the risk factors for the development of the prostate cancer. Treatment failure of the prostate cancer can result into the castration resistant prostate cancer that further can become problematic and untreatable^1^. Prostate cancer become aggressive and become resistant to therapies leading to the activation of various tumor progression signals and can induce chemo, radio and immune resistance. The understanding of prostate cancer biology has evolved considerably in recent years. It is now widely acknowledged that genetic alterations play a crucial role in the initiation and progression of prostate cancer. Genomic studies have identified several key genetic aberrations, such as alterations in the androgen receptor (AR) signaling pathway, TMPRSS2-ERG fusion, and mutations in tumor suppressor genes, which contribute to the development and aggressiveness of prostate cancer^2^. Furthermore, the identification of specific molecular subtypes of prostate cancer has enabled a more personalized approach to treatment, tailoring therapies based on the genetic profile of the tumor^3^.

β-glucan has been widely recognized for its immuno-modulatory effects. Recent studies have shown that β-glucan can enhance the immune response by activating immune cells, such as macrophages and natural killer cells, and promoting the production of cytokines and antibodies^4,5^. These immune-enhancing properties make beta glucan a promising candidate for improving immune function and supporting immune-related conditions, such as infections, allergies, and certain types of cancer. Furthermore, research suggests that beta glucan may have potential cardiovascular benefits, including cholesterol-lowering effects and the improvement of lipid profiles^6^. Such findings have sparked interest in exploring the applications of beta glucan as a dietary supplement and functional ingredient in food products. Quercetin, known for its antioxidant and anti-inflammatory properties, has attracted attention in various areas of research. Recent studies have revealed its potential in mitigating oxidative stress and reducing inflammation, which are key factors associated with chronic diseases, including cardiovascular disorders, neurodegenerative conditions, and certain types of cancer^7^. Quercetin has also been investigated for its antiviral properties, particularly its ability to inhibit viral replication and modulate immune responses, which has garnered attention during the COVID-19 pandemic^8^. Additionally, emerging research suggests that quercetin may have beneficial effects on metabolic health, such as glucose regulation and insulin sensitivity^9^. Quercetin is reported to have efficient anticancer activity against prostate cancer which is validated through various in vitro, in vivo studies. In a research, quercetin induced apoptosis in DU145 cells through the enhanced tumor necrosis factor-related apoptosis-inducing ligand that resulted in overexpression of death receptor 5. A study demonstrated its anticancer activity against prostate cancer in vivo in which researchers used severe combined immunodeficiency mice for the androgen-sensitive LAPC-4 xenograft prostate tumor. Here quercetin targeted AKT/mTOR pathway and was found to reduce the poundage and volume of the PC3 tumor in 6-week-old BALB/c nude mice^10^. Moreover, quercetin-loaded PLGA nanoparticles were found to have increased cytotoxicity and cellular uptake compared to the no-targeted delivery system on LNCaP cells due to the enhanced prostate specific membrane antigen activity^11^.

These diverse therapeutic potentials have positioned quercetin as a promising natural compound for preventive and therapeutic applications still there are various drawbacks that lead to the poor systemic availability of the quercetin. Quercetin is not soluble in water, have poor bioavailability and metabolized rapidly in the body which are limiting factors for the use of quercetin as an treatment option. Its encapsulation in any biocompatible and biodegradable nano/microparticles formulation may lead to it long term retention in blood along with delayed metabolism^12^. A study developed quercetin loaded nano-encapsulation using zein, shellac, and chitosan. Encapsulation efficiency was 74.95% when mass ratio of chitosan and zein was kept 1:4 and maximum release amount was 59.62% for this formulation^13^. Although, multiple studies used nano-formulations for the loading of polyphenols in the dietary fibers such as β-glucan but still there is much unknown about the interaction between them. A very few studies have discussed about it such as research focused on the adsorption of the derivatives of quercetin onto the β-glucan. Obtained isotherms with improvement in regression showed that quercetin-3-rhamnoside and quercetin-3-glucoside were highly adsorbed on the β-glucan than the quercetin-3-galactoside. This is also hypothesized in this study that –OH group’s arrangement in the sugar present in the quercetin derivatives had an effect on the adsorption capacity^14^. One more study showed that hydrogen bond could be formed between derivatives of quercetin and β-glucan due to their –OH groups that can lead to the formation of van der waals interaction due to the shortening of the distance between the β-glucan and polyphenol molecule^15^. Accurate and timely diagnosis of prostate cancer is vital for effective management. Traditional diagnostic methods, such as digital rectal examination and prostate-specific antigen (PSA) testing, have limitations in terms of specificity and sensitivity. However, recent advances have shown promise in improving prostate cancer detection. Search for treatment strategy with some bioactive compounds can lead to an efficient cure for the prostate cancer. In this study we have discussed the loading of quercetin in β-glucan, and estimated their stability using TGA and DSC techniques. We reported slowed quercetin release from β-glucan after alginate sealing and found the formulation to show efficient anticancer activity against prostate cancer cell line PC3.

## Materials and methods

### Reagents and chemicals

Ammonium Buffer Solution, Hydrochloric acid, Lead (II) acetate trihydrate, Ninhydrin, Fehling A and B solutions, Potassium ferricyanide, trichloroacetic acid, and DPPH (2, 2-Diphenyl-1-picrylhydrazyl) were purchased from HiMedia, MTT, DAPI, PI, H2DCFDA, LysoTracker Red DND 99, MitoTracker Red CMX-ROS, Acridine orange, Ethidium bromide were purchased from Invitrogen (Thermo Fisher Scientific).

### Cell culture and maintenance

The prostate cancer cell line PC3 was purchased from the National Centre for Cell Science (NCCS) in Pune, India. All cell culture products, including RPMI media, fetal bovine serum (FBS), antibiotic, and antimycotic solution, were purchased from Gibco™ (Thermo Fisher Scientific). Cells were maintained in a humidified environment at 37 °C and 5% CO_2_ supply. RPMI was supplemented with 10% FBS and 1% antibiotic and antimycotic solution.

### Particle preparation

#### Preparation of the β-glucan particles

β-glucan particles from *Saccharomyces cerevisiae* and *Agaricus bisporus* were prepared according to the previously published protocol and particle preparation from *Saccharomyces cerevisiae* which we have already published in our previous study^16^. For the particle preparation from *Agaricus bisporus*, 100 g of fruiting bodies (FBs) was taken and washed three times with double distilled water (DDW). FBs were then crushed and suspended in 200 ml of DDW and boiled in a heating mental up to 30 min. Boiled FBs were then centrifuged at 9000 rpm for 10 min and washed with 80% of the ethanol. Further, the pellet was added in 100 ml of DDW and kept in a water bath for 4 h at 90 °C temperature. Boiled material centrifuged at 9000 rpm for 10 min and pellet was added in 1 M NaOH and kept in water bath for 1 h at 70 °C. Pellet is collected by centrifugation at 9000 rpm for 5 min and suspended in 100 ml DDW to adjust pH at 4–5 with the help of Hydrochloric acid. pH adjusted suspension kept in a water bath for 30 min at 70 °C and centrifuged at 9000 rpm for 10 min. Collected pellet was vortexed and washed twice with Isopropyl alcohol (IPA) at 9000 rpm for 15 min. Furthermore, pellet was washed twice with acetone at 9000 rpm for 15 min and lyophilized to collect particles^17,18^. In this study, β-glucan particles prepared from yeast and *Agaricus bisporus* were named as YBG and ABG, respectively.

#### Loading of Quercetin in prepared β-glucan particles and their alginate sealing

For the Quercetin loading and alginate sealing, we referred to a previously published protocol^19^. Quercetin stock solution was prepared as 500 mg/10 ml of 0.2 N Hcl. 500 mg Quercetin and 500 mg β-glucan (1:1 ratio) (ABG and YBG) were mixed to make a uniform paste and incubated at room temperature (RT) for 2 h. Further, 1 M Tris buffer (pH 8) was added and the precipitate was collected by centrifuge, and lyophilized. Here we named hollow β-glucan particle A1 and Y1 for ABG and YBG, Quercetin as Q and A2 and Y2 for the Quercetin-loaded ABG and YBG particles respectively. Quercetin loaded lyophilized particles were further alginate sealed to seal the pores of the β-glucan for the slow and sustained release of the Quercetin from β-glucan. 200 mg of the each A2 and Y2 particles were taken and dissolved in 0.25% sodium alginate and incubated at RT for 1 h. Particles were centrifuged at 9000 rpm for 5 min and the whole process was repeated. Further, 2% CaCl_2_ was added for the cross linking and particles were incubated over night at RT. Incubated particles were centrifuged at 9000 rpm for 5 min, collected and lyophilized. Particles were named as A3 and Y3 for the Quercetin loaded alginate sealed particles prepared from *Agaricus bisporus* and Quercetin loaded alginate sealed particles prepared from yeast respectively.

### Quantification of the prepared β-glucan

Quantification of the prepared β-glucan from yeast and *Agaricus bisporus* by Anthrone test and HPTLC analysis was already published in our previous studies^20,21^.

#### Quantification by aniline blue staining

Aniline blue staining was performed according to a previously described protocol for the quantification of the β-glucan. For the analysis, 50, 100, 150, 200 and 250 µg/ml concentrations were made in 1 M NaOH and 300 µl of this sample was taken and 6 M NaOH was added. Further, the sample was heated at 80 °C for the 30 min in a water bath for the unbranching of the triple helical β-glucan. After the unbranching of triple helical structure, the sample was incubated at 50 °C for the 30 min and 630 µl of aniline blue sample (21 parts 1 mol/L hydrochloric acid, 59 parts 1 mol/L glycine buffer at pH 9.0, 40 parts aniline blue solution 0.1% in water) was added and the sample was further incubated at RT for the 30 min. The fluorescence of the sample was measured at excitation and emission wavelengths 400 and 500 nm respectively^22^.

#### Quantification by Congo red staining

Congo red is specific for the quantification of the triple helical structure of the β-glucan. We performed this experiment according to the previously described protocol^23^. The β-glucan with different concentrations was dissolved in 0.5 M NaOH 24.5 µM Congo red dye was added, and spectra were recorded after scanning from 300 to 700 nm wavelength.

### Characterization of the prepared particles

#### Size and zeta potential analysis

Prepared particles A1, A2, A3, and Y1, Y2, Y3 were analyzed using Malvern Zeta sizer v2.2. This instrument works on the DLS (dynamic light scattering) principle and measures the particle size, PDI (polydispersity index) value and zeta potential^24^.

#### Fourier transform infra-red spectroscopy

FTIR analysis was performed for the analysis of functional groups present in the β-glucan and Quercetin loaded β-glucan to confirm the nature of the particles those were extracted with the acid base extraction method^25^.

#### Morphological analysis with scanning *electron* microscope (SEM) and energy dispersive X-ray (EDX) analysis

Morphology of the prepared particles was analyzed with the help of FESEM (field emission scanning electron microscopy) and Energy dispersive x-ray spectroscopy (EDS) was performed the elemental analysis of the particles^26^.

#### XRD analysis to determine the nature of particles

To determine whether the particles are crystalline or amorphous, x ray diffraction analysis was performed. Diffraction intensities were recorded from 10° to 60° 2́θ^27^

#### Thermogravimetric analysis (TGA)

TGA analysis was performed to determine the weight loss of the sample with respect to temperature^28^. Samples were heated in the range of 50 °C to 750 °C at 10 °C/min, switched the Gas to Nitrogen at 20 ml/min and hold for 1 min at 750 °C.

#### Differential scanning calorimetry analysis (DSC)

DSC analysis was performed to measure the thermal properties of the prepared samples. For the DSC analysis, samples were heated from 30 to 440 °C at 10 °C/min and held for 1 min at 440 °C^29^.

### In vitro* drug release profile*

In vitro drug release test was carried out as per previously described protocol^30^. Briefly, 1 mg of each sample was added into the 100 ml of PBS (pH 7.4) and acetate buffer (pH 5.2), and each suspension was kept in a shaker incubator at 100 rpm and 37 °C temperature. 3 ml aliquot of the samples was taken at time periods of 1, 2, 4, 6, 8 and 24 h and 3 ml of fresh buffer was added to maintain the sink conditions. The amount of the released drug was determined with the absorbance that was taken at 290 nm with the help of a UV spectrophotometer.

### Determination of hemolytic inhibition

To determine the hemolytic effect of the prepared formulation against human erythrocytes, as per the previous protocol with slight modifications^31^. Briefly, 5 ml of the blood was centrifuged and diluted with PBS (pH 7.4). 200 µl of the diluted blood was mixed with the 200 µl of the samples (prepared in incomplete RPMI) and incubated for 24 h at 37 °C temperature. Triton 100X was used as a control for the 100% lysis of RBCs. Absorbance was measured at 450 nm with the help of BioTek Synergy H1 Multimode Reader. % hemolysis was calculated using given formula:

$$\text{\% hemolytic activity}=\frac{(\text{Absorbance of the Control}-\text{Absorbance of the sample})}{\text{Absorbance of the control}}\text{ X }100$$

### Antioxidant activity determination

#### DPPH analysis

DPPH is a free radical and it is found to have efficient radical scavenging activity. The assay was performed as per the previously reported protocol^32^. DPPH solution was made by dissolving 0.1 mM of DPPH in methanol, and 2 ml of it was added in the different concentrations of the prepared samples. Samples were incubated for 30 min in the dark and absorbance was measured at 517 nm with the help of a UV–vis spectrophotometer. % Radical scavenging activity was calculated using following equation:

$$\text{\%RSA}=\frac{\text{Absorbance of control}-\text{Absorbance of sample}}{\text{Absorbance of control}}\text{x}100$$

#### Reducing power assay

Reducing power is an indication of the antioxidant activity of any compound. Reducing power was determined according to the previously described protocol^32^. 2.5 ml of the various concentrations of the samples were taken, and mixed with 2.5 mL of 1% potassium ferricyanide and 2.5 ml of 0.2 M sodium phosphate buffer (pH 6.6). Mixture was incubated at 50 °C for the 20 min. After incubation, 2.5 ml of 10% trichloroacetic acid was added and the mixture was centrifuged at 2000 g for 10 min. The upper 5 ml layer was mixed with 5 ml of DDW and 1 ml of 0.1% ferric chloride and the absorbance was measured at 700 nm.

#### Determination of the total phenolic content (TPC)

Total Phenolic content was determined with Folin-Ciocalteu (FC) colorimetric assay by previously reported protocol^33^. Gallic was taken as standard and 300 µg/ml of the sample was taken to determine its total Phenolic content. 25 µl of each standard and sample were mixed with the 125μL of FC reagent and incubated for 10 min at RT. Neutralization of the reaction was performed by adding 125μL of 20% Na2CO3 solution and further incubated for 1 h at RT and absorbance was measured at 760 nm. TPC was calculated as equivalent per gram of Gallic acid.

### Determination of anticancer activity

#### Assessment of the cell viability with MTT assay

Cell viability of the prepared particles was determined with the help of MTT assay as per the previously described protocol^34^. Briefly, 10,000 cells per well were seeded in a 96 well plate and incubated for 24 h in a humid environment with 5% CO_2_ and 37 °C temperature. After incubation cells were treated with the prepared particles in serial dilution from the 500 µg/ml for 24 h. After 24 h, old media was decanted and 10 µl of the MTT dye was added to each well further incubated for 4 h at 37 °C. After incubation was over, 100 µl of the DMSO was added in each well and kept for 10 min to solubilize the formazan crystals. Absorbance of the dissolved formazan crystals was measured at 490 nm and % cell viability was calculated with the given formula:

$$\text{\%Viability}=\left(\frac{\text{Absorbance of treatment}}{\text{Absorbance of control}}\right)\text{X}100$$

Concentrations of IC_50_ and below and above IC_50_ were selected for the further analysis.

#### Morphological observations

Morphological changes of the treated cells were observed according to a previously described protocol^35^. In brief, 50,000 cells/well were seeded in 24-well plate and left overnight for attachment. After 24 h, cells were treated with the selected doses of the particles and Quercetin and further incubated for 24 h at 37 °C and 5% CO_2_. After incubation was over, cells were washed with PBS and morphological changes were observed and captured with the help of fluorescence microscopy (EVOS FLoid imaging station).

#### Reactive oxygen species (ROS) generation

ROS generation after treatment with the particles was determined using the previously described protocol^36^. Briefly, 50,000 cells/well were seeded in 24 well plate and left for 24 h for incubation and attachment. After 24 h, cells were treated with selected doses of all the particles and Quercetin and kept for 24 h incubation at 37 °C and 5% CO_2_. After incubation, cells were washed with PBS, and stained with 20 µM DCFDA dye and kept at incubation in 37 °C for 20 min and images were captured with EVOS FLoid imaging station.

#### Determination of nuclear morphology and DNA fragmentation with DAPI staining

DAPI staining was performed with the help of previously described protocol with slight modifications^37^. A total of 50,000 cells/well were seeded in the 24 well plate and after 24 h of incubation treated with various concentrations of the test compound and kept for incubation at 37 °C and 5% CO_2_ for another 24 h. After incubation was over, cells were washed with PBS and fixed with chilled methanol for 10 min. After fixation, the cells were washed with PBS and stained with 1 μg/mL of DAPI for 15 min. Morphological changes were observed and photographed.

#### Apoptosis detection with Propidium iodide (PI) staining

Apoptosis of the treated cells was determined with the help of PI staining with the help of previously described protocol with slight modification^38^. In Brief, 50,000 cells per well were seeded in a 24 well plate and incubated overnight. After incubation, cells were treated with selected concentration of Quercetin and prepared particles and incubated at 37 °C and 5% CO_2_ in a CO_2_ incubator for 24 h. After incubation, cells were stained with 1 µg/ml of the PI (prepared from 1 mg/ml of PI) and further incubated for 10 min at 37 °C. After incubation, images were obtained with the EVOS FLoid imaging station.

#### Acidic organelles activity analysis through LysoTracker red DND-99

Acidic organelles were detected with the help of LysoTracker Red (100 nM) as per the previously described protocol^38^. Briefly, 50,000 cells/well seeded in 24 well plate and incubated overnight. After attachment of the cells, treatment with the various concentrations of particles was performed and the cells were further incubated for 24 h. After incubation, cells were stained with LysoTracker for 30 min and imaged using an EVOS FLoid imaging station.

#### Estimation of mitochondrial membrane potential through MitoTracker red CMX-ROS

Mitochondrial membrane potential was assessed as per the previously reported protocol^39^. Briefly, 50,000 cells/well seeded in 24-well plate and incubated for 24 h. After incubation, cells were treated with various concentrations of the particles and further incubated for 24 h. After incubation, cells were stained with MitoTracker red CMX ROS (300 nM) for 30 min and imaged using an EVOS FLoid imaging microscope.

#### Early and late apoptosis detection with Acridine orange and Ethidium bromide (AO/EtBr) staining

AO/EtBr dual staining was performed according to a previously reported protocol with slight modification^40^. A total of 50,000 cells/per well seeded in 24well plate and after confluency, treated with various concentrations of particles and left for 24 h incubation in CO_2_ incubator. After incubation, cells were stained with 5 µl of AO and 5 µl EtBr (5 mg/ml each) and cell death was observed with the help of FLoid Imaging microscope for morphological changes.

### Cell migration/scratch assay

Scratch assay was performed according to a previously described protocol^41^. Briefly 50,000 cells/well were seeded in a 6-well plate and left for incubation until a complete monolayer was formed. After incubation, a scratch was made using 200 µl of pipette and cells were imaged under LABOMED-TMC400 inverted microscope. After imaging, cells were treated with IC50 concentration of different particles and left for the 24 h incubation. Cells were again imaged after the incubation period is over. % Area of the scratch or wound was quantified by the given formulae^42^.

$$\text{Wound area \%}=\frac{\text{At}}{\text{A}0}\text{x}100$$

where A_t_ = wound area after treatment and A_0_ = Wound area before treatment.

### DNA fragmentation assay

DNA fragmentation assay was performed according to the given protocol^43^. 1 × 10^5^ cells/ well were seeded in 6 well plate and left overnight for the growth and attachment. Next day, cells were treated with IC_50_ concentrations of A2, A3 and Y2, Y3 particles and incubated for 24 h. After incubation, floating cells of media (apoptotic cells) and cells attached on the well were collected and DNA was extracted with the chloroform-Isoamyl alcohol method. Electrophoresis was carried out in 1% agar and bands were visualized using a Gel documentation system (Thermo Fisher Scientific).

### Statistical analysis

All of the cell culture and antioxidant activity experiments were performed in triplicates. One-way ANOVA was applied using GraphPad Prism 8.0 and ImageJ. Graphs were made using GraphPad Prism and OriginPro software. A probability value of *p* < 0.05 was considered statistically significant where *** means highly significant, ** means less significant and * means least significant. All the data were expressed as the mean ± standard deviation of the mean (S.D.) from triplicate experiments.

## Results

### Particle preparation

#### Prepared particles were β-glucan

**Figure 1:**
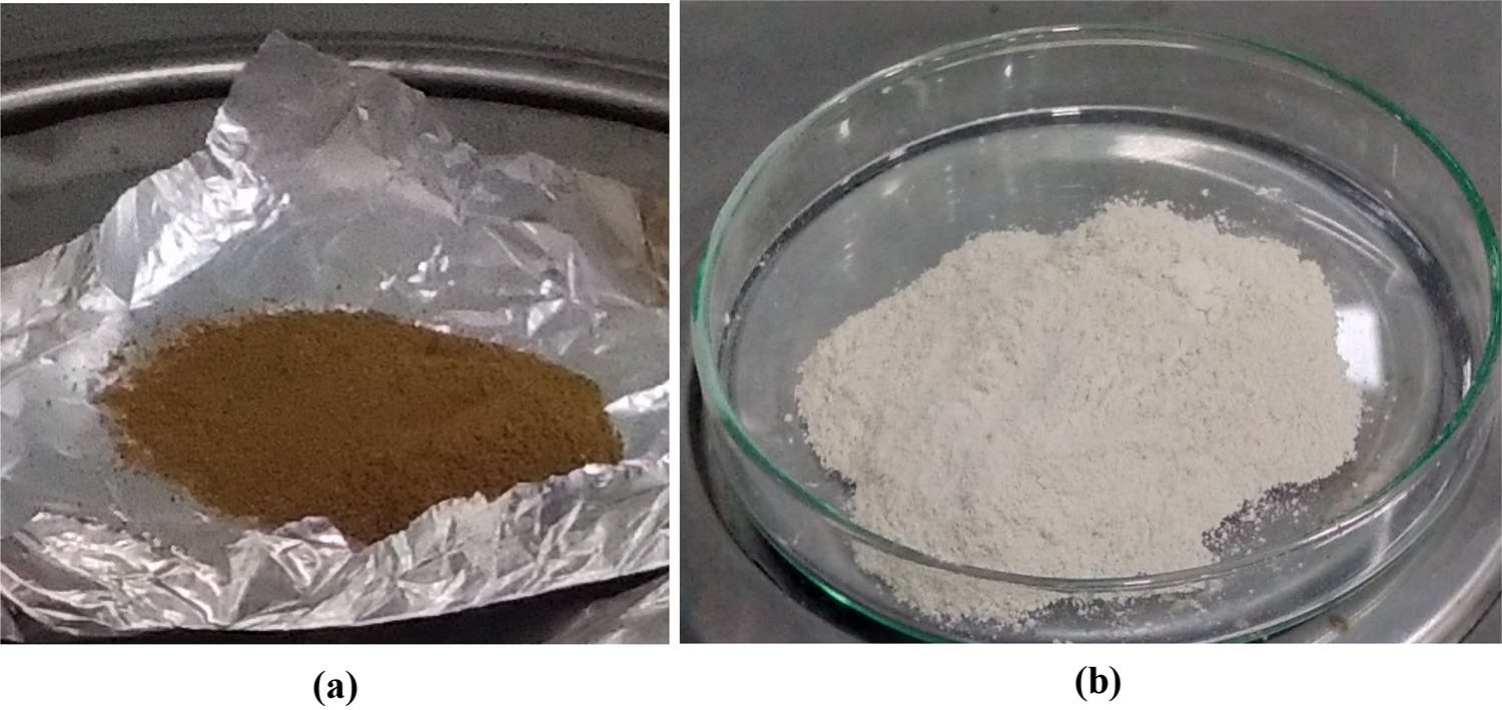
Prepared β-glucan particles. (**a**) β-glucan from *Agaricus bisporus* having slightly brown color. (**b**) Yeast β-glucan having white color powder.

Prepared particles from the yeast and *Agaricus bisporus* were white and brown in color respectively as shown in Fig. 1.

#### Loading of Quercetin in prepared β-glucan particles and their alginate sealing

**Figure 2:**
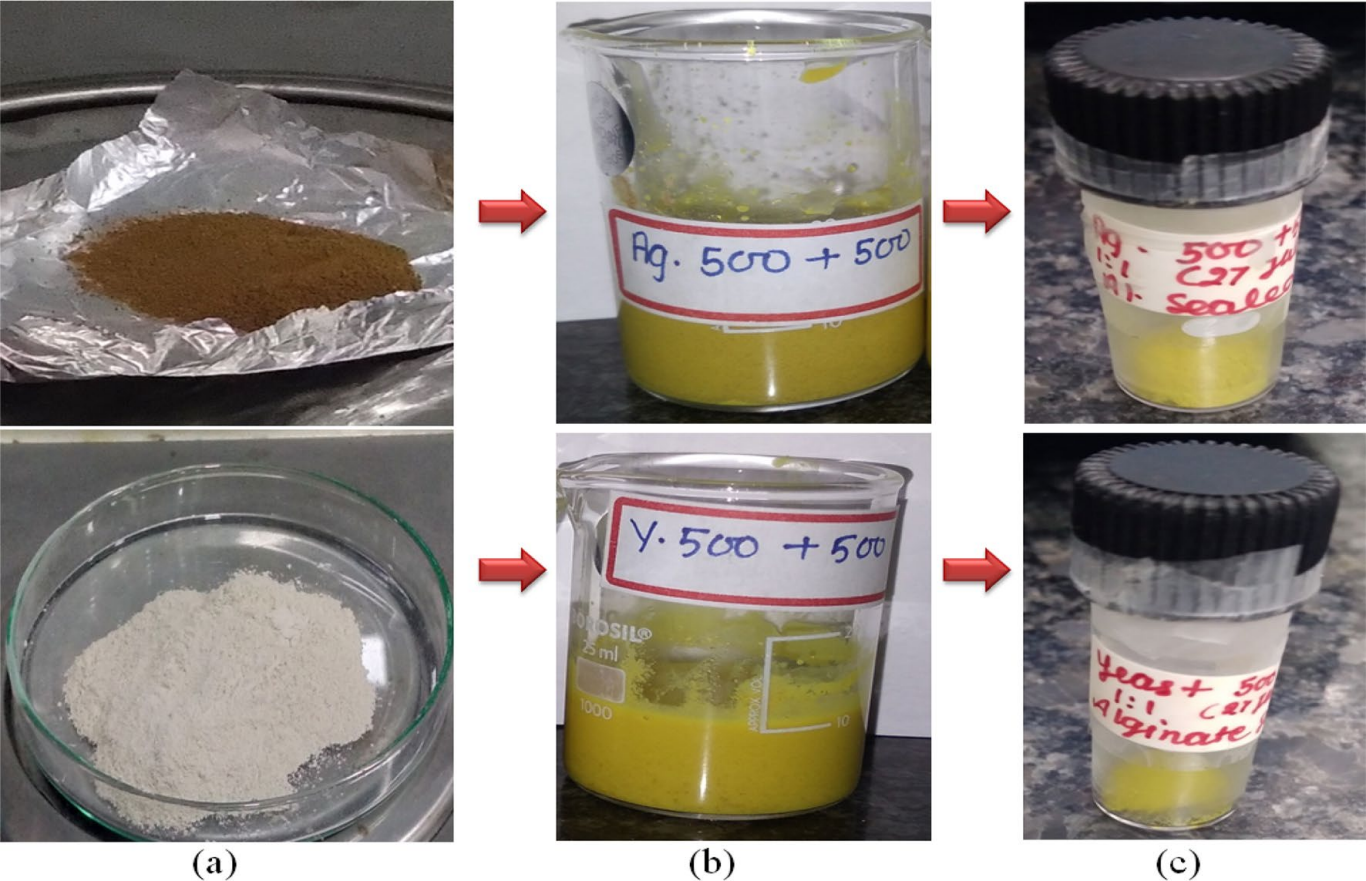
Hollow β-glucan particles (**a**) were loaded with the Quercetin as shown in (**b**). (**c**) Quercetin loaded particles were sealed with the sodium alginate.

500 mg Quercetin and β-glucan each were taken for the loading and prepared particles were lyophilized. Lyophilized particles were sealed with sodium alginate. CaCl_2_ was used for the cross linking with the sodium alginate for the proper sealing. This sealing was performed for the slow and sustained release of the Quercetin from the hollow β-glucan particles. Yellow colored particles were obtained after lyophilization as shown in Fig. 2.

### Quantification of the prepared β-glucan

#### Quantification by aniline blue staining dye to confirm the presence of β-1, 3 linkage in prepared β-glucan formulation

**Figure 3:**
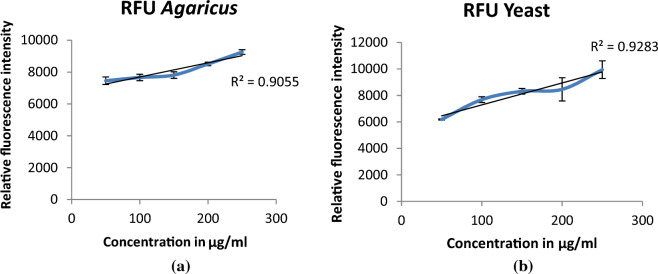
Breakdown of the triple helical structure into single helical structure resulted in increased RFU with increased concentration of the β-glucan from (**a**) *Agaricus bisporus* and (**b**) yeast.

Aniline blue dye specifically binds to the single helical structure of the β-1, 3 Glucan resulting in the fluorescence emission. In our study, Relative fluorescence intensity (RFU) was found to increase as shown in Fig. 3 with the increase in concentration of the β-glucan thereby confirming the presence of the β-1,3 linkages in the extracted β-glucan from the yeast and *Agaricus bisporus*.

#### Quantification by Congo red staining to confirmed the triple helical structure of the β-glucan

**Figure 4:**
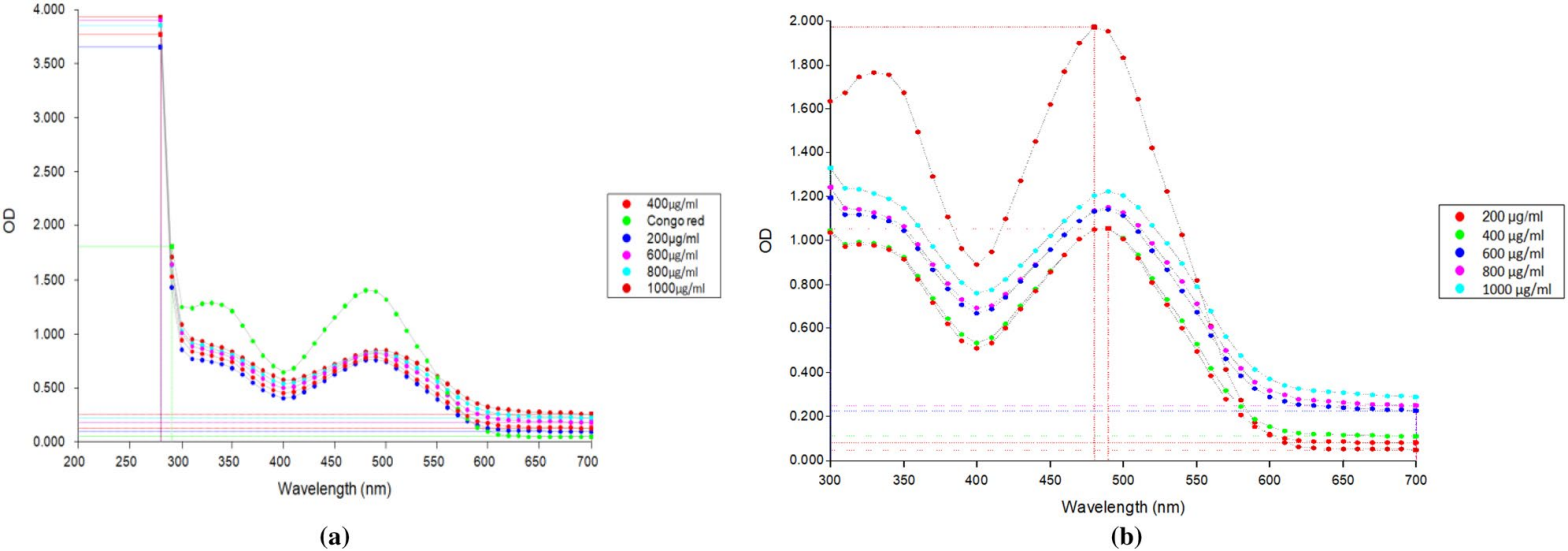
A bathochromic shift in isolated β-glucan with the increase in concentration of the β-glucan confirming the triple helical structure of β-glucan with β-1, 3–1, 6 branching.

Congo red staining quantifies the triple helical 1,3–1,6 structure of β-glucan. A continuous bathochromic shift with increase in concentration was observed for the β-glucan isolated from the *Agaricus bisporus* and yeast as shown in Fig. 4. This bathochromic shift confirms that β-glucan is having β-1, 3–1, 6 linkage and triple helical structure as Congo red does not binds with single helical β-1,3 glucan^44^.

## Characterization of the prepared particles

### Size and zeta potential analysis

**Figure 5:**
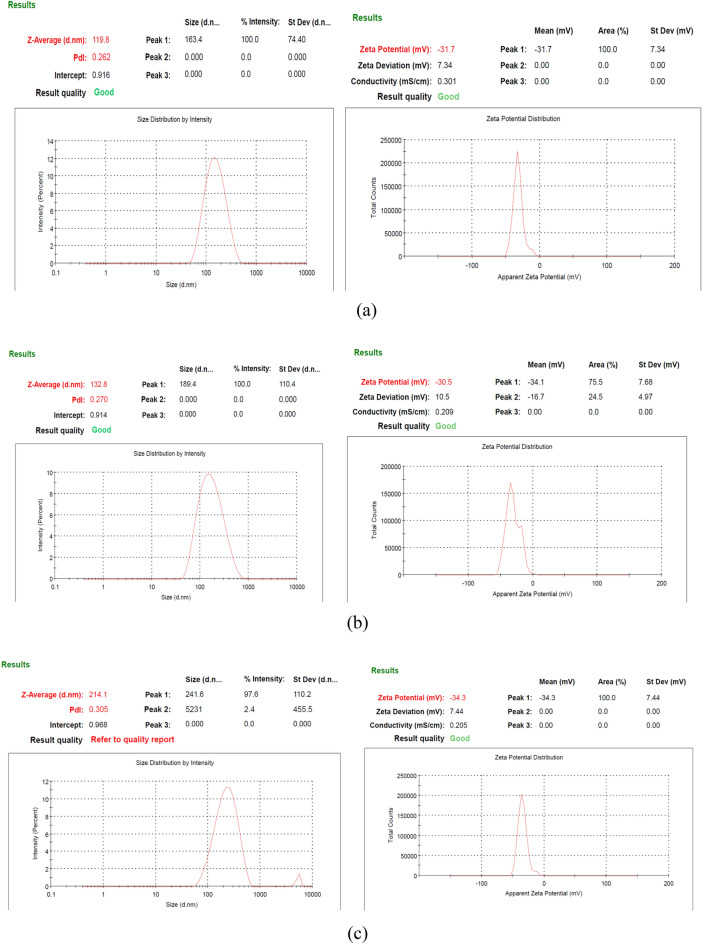

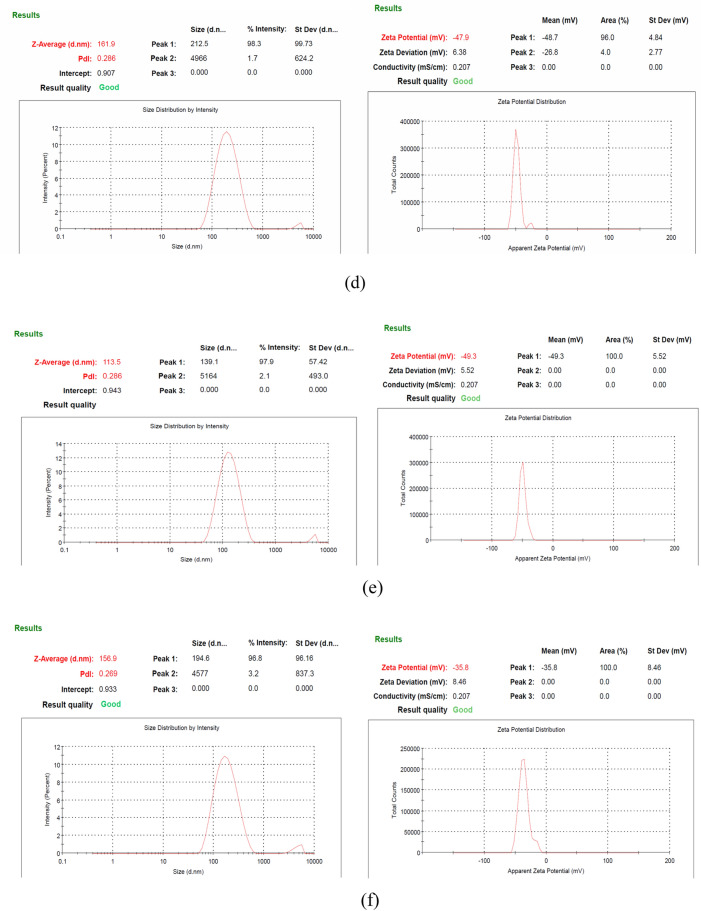
Size and zeta potential analysis of the prepared particles. (**a**, **b**, **c**) are the size and the zeta potential of the A1, A2 and A3 respectively while (**d**, **e**, **f**) are the size and zeta potential of the Y1, Y2 and Y3 respectively.

Loading of Quercetin and further alginate coating in the β-glucan has increased the size of the particles but there was a drop in the zeta potential confirming the successful alginate coating. Although all the particles were in the range of 100–250 nm of size and are suitable for the uptake by the cells as shown in Fig. 5. In some studies, suitable size of the particles for the cellular uptake investigated and it is found that particles less than 200 nm of the diameter are internalized into the cell through the clathrin-coated pits while particles of the size about 500 nm involve the use of caveolae-mediated internalization mechanism^45^. After alginate coating although the size was increased but the zeta potential decreased that is the indication that negatively charged sodium alginate interacted electrostatically with the β-glucan particles^46^. Although the zeta potential of the Y3 particles was found to be increased after alginate sealing. All the particles have a PDI (Polydispersity index) value between 0.2 and 0.3 that is clear indication of the homogeneity of the particles.

#### Fourier transform infra-red spectroscopy

**Figure 6:**
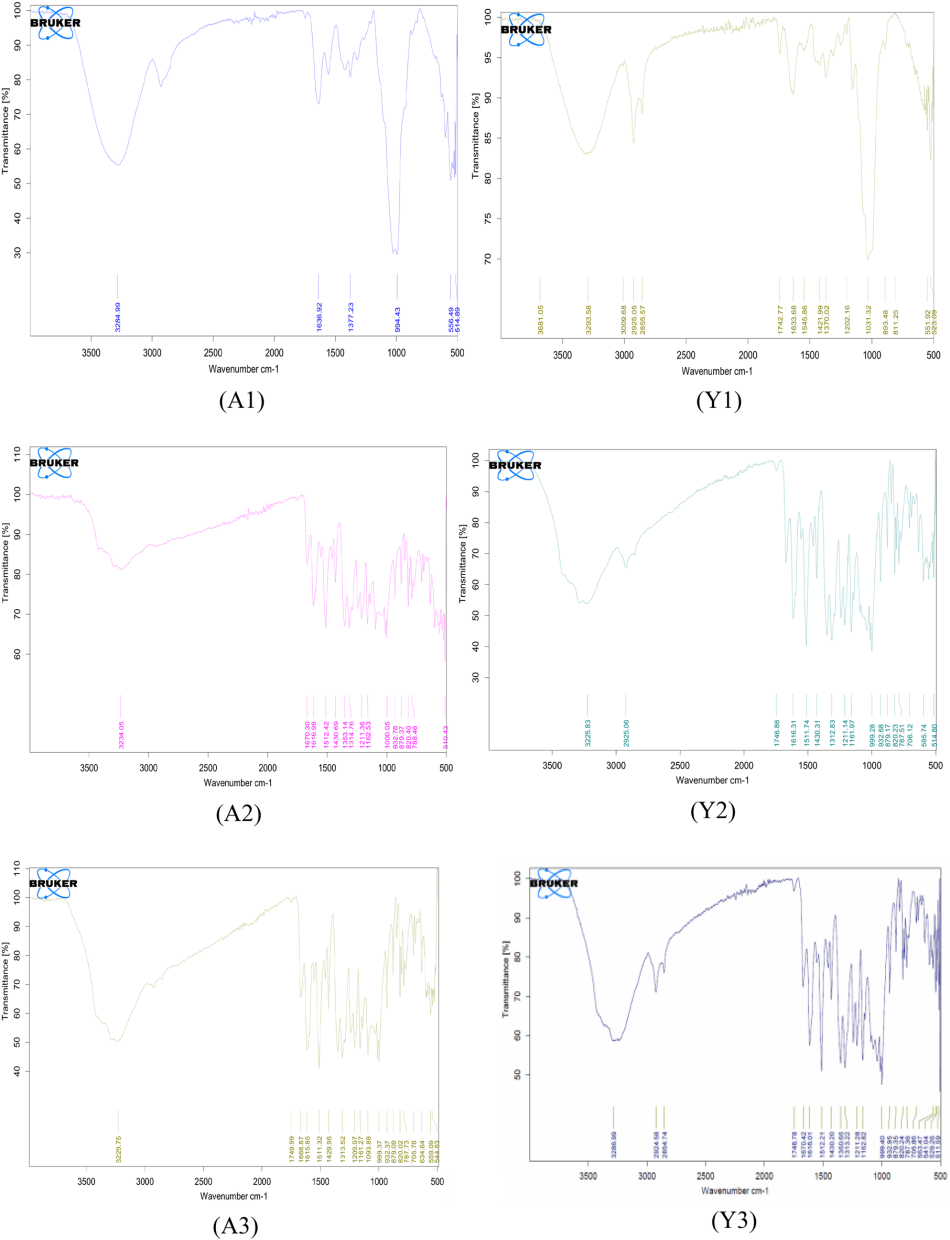
FTIR analysis of the hollow β-glucan particles, Quercetin loaded and alginate sealed particles.

FTIR is used for the functional group analysis of the compound. In general peaks at wavenumbers (Cm^−1^) 890, 1076, 1372, 2919, and 3390 are responsible for β-1,3 linkage, CO stretch, CHOH stretch, CH stretch, and OH stretch respectively^47^. In our isolated β-glucan, major peaks were present at 893, 1031, 1370, 2925, 3293 as shown in Fig. 6. A1 and Y1, confirming the isolated compound is β-glucan. For the Quercetin, major peaks are at wavenumbers 3292, 1663, 1508, 1354, 1255–1000 for the phenolic OH stretching, CO stretching, CC stretching, C–OH deformation, and COC anti-symmetrical and symmetrical stretching respectively^48^. In the A2 and Y2 Quercetin loaded β-glucan showed peaks for the both i.e., Quercetin and β-glucan. Major peaks were reported at around the wavenumbers 3234, 1670, 1512, 1353, 1211, 1162, 1000, and 879 that were almost same as for the Quercetin and β-glucan. Sodium alginate has its characteristic peaks at wavenumbers 1608, 1420, and 1034 for the anti-symmetric stretching vibration of –COO–, symmetrical stretching vibration, and stretching vibration of C–O–C respectively^49^. Alginate sealed particles were showing these peaks with the little numeric variation in A3 and Y3 indicating the presence of alginate coating around the particles.

#### Morphological analysis with scanning *electron* microscope (SEM) and energy dispersive X-ray (EDX) analysis

**Figure 7:**
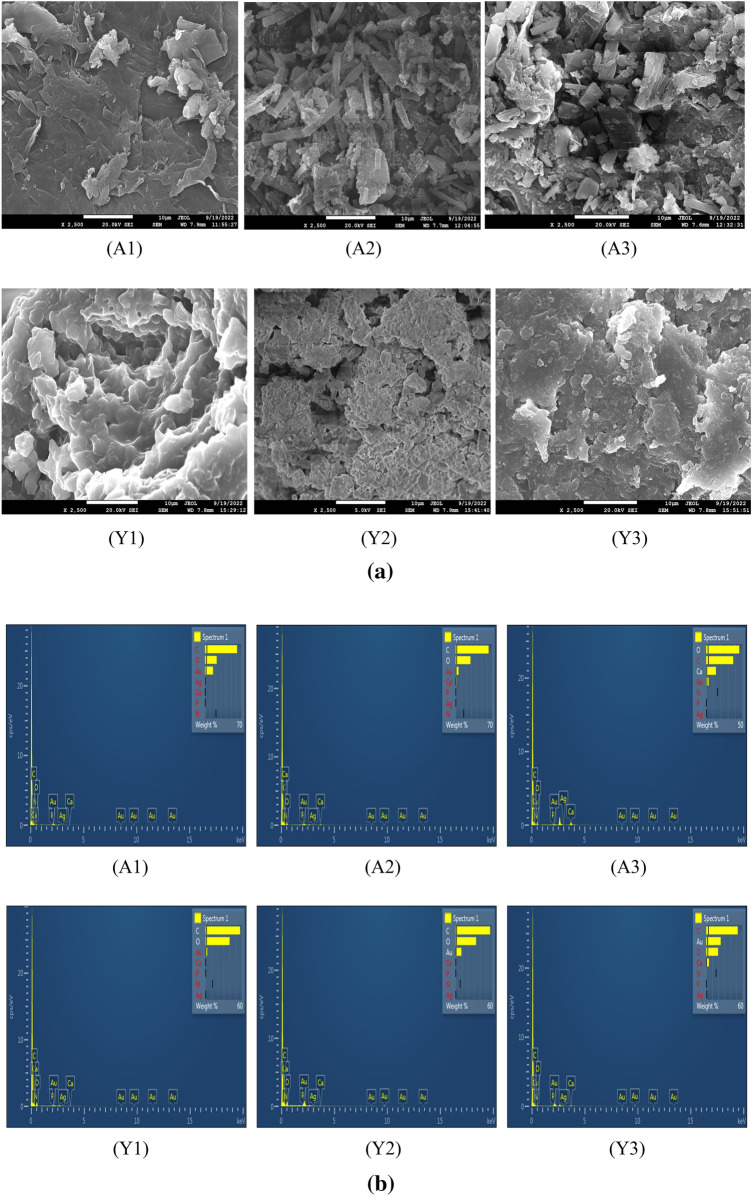
(**a**) Surface morphology of the prepared particles. (**b**) Elemental analysis with the help of EDX.

*Agaricus bisporus* derived particles were elongated in shape while yeast derived particles were little bit spherical in shape as shown in Fig. 7a. Surfaces were not exactly smooth. A study by computer simulation found that elastic or soft particles are less efficient for the uptake by the cells while rigid particles can be efficiently internalized. Rigidity of the particles provides high energy barriers for the wrapping of the membrane while soft particles need more receptors to overcome this energy barrier^50^. EDX spectra and present elements are shown in Fig. 7b and Table 1 respectively. Average size from the SEM analysis of particles was evaluated using the ImageJ software. The average size of the A1, A2, and A3 was 8.04, 8.09 and 9.15 respectively while average size of the Y1, Y2, and Y3 was 9.14, 3.32, and 3.93 respectively. Size estimated from ImageJ was in µm and scale was set at 10 µm.

**Table 1:** Elementals analysis of the particles.

Element	Wt%					
	A1	A2	A3	Y1	Y2	Y3
C	61.69	63.51	37.36	57.08	56.89	52.08
N	0.00	0.00	0.00	0.00	0.00	0.00
O	22.42	28.47	45.66	39.74	33.74	19.58
P	0.00	0.60	0.00	0.15	0.02	0.00
Ca	0.18	1.41	13.49	0.25	0.08	4.44
Ag	0.32	0.51	0.00	0.00	0.00	0.00
Au	15.39	5.50	3.49	2.78	9.27	23.91
Total	100.00	100.00	100.00	100.00	100.00	100.00

#### XRD analysis confirmed that particles are crystalline in nature

**Figure 8:**
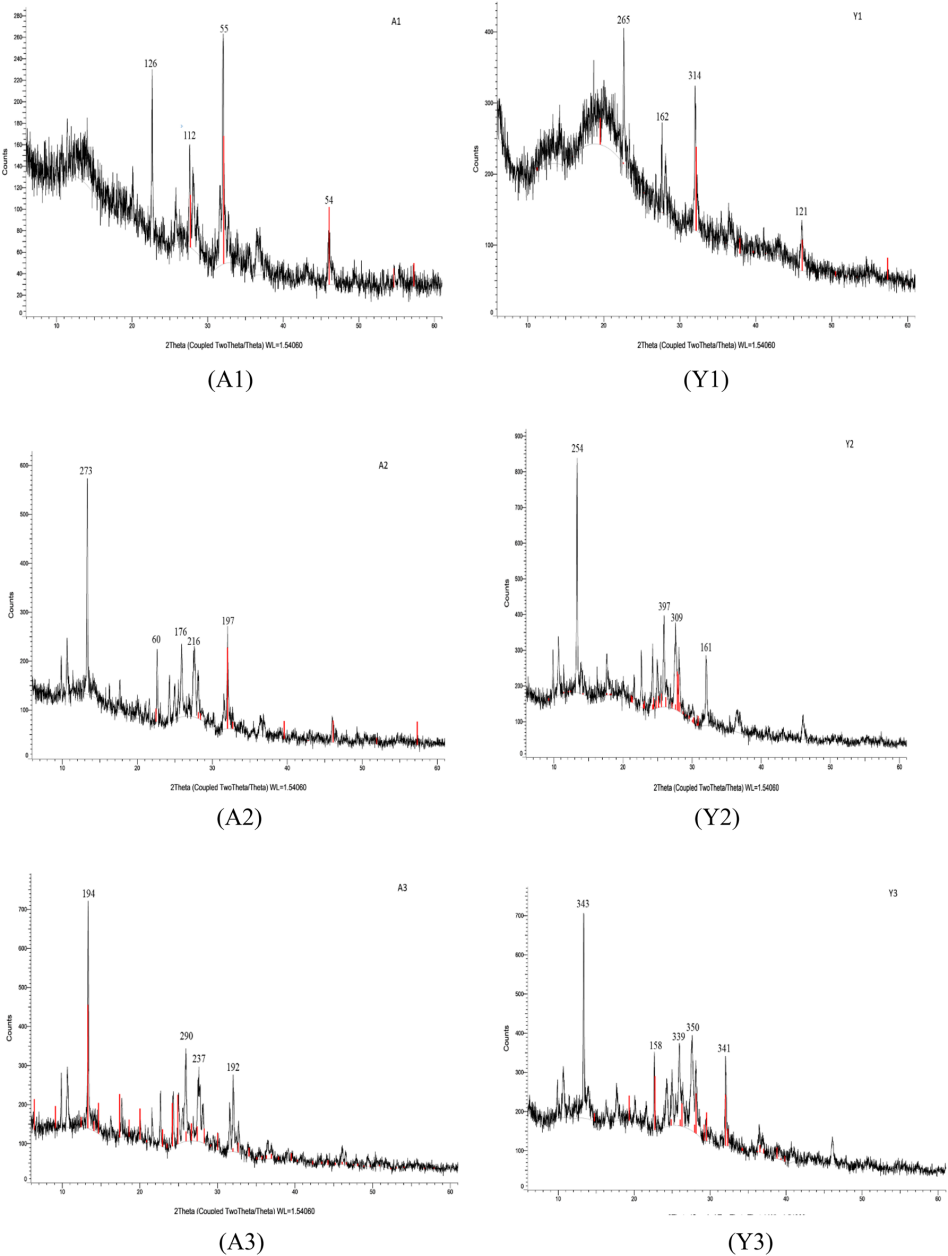
XRD analysis of the prepared particles showing that all the prepared particles were crystalline in nature.

In XRD analysis, all the particles were found to have a clear narrow peak sowing crystalline nature of the all the particles as shown in Fig. 8. A sharp peak between 2θ 20 and 30 is indicative of β-glucan which was present in both A1 and Y1. Quercetin is found to have its reported peak between 10 and 15 2θ and this peak was present in quercetin loaded β-glucan indicating that quercetin is successfully entrapped inside the β-glucan. Froom the spectra, it is evident that formulation is more crystalline in nature than hollow β-glucan particles.

#### Thermo gravimetric analysis (TGA)

**Figure 9:**
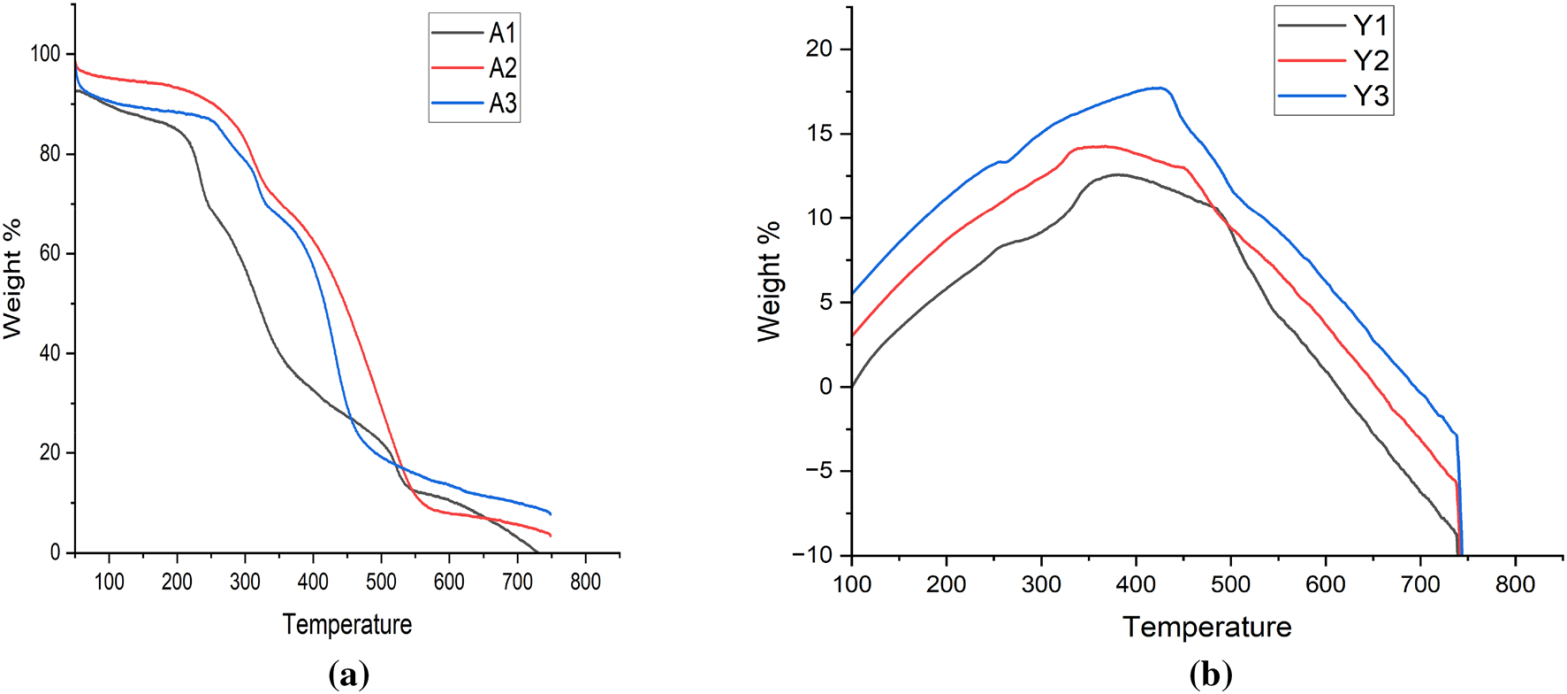
TGA analysis of the prepared particles. (i) Analysis of the particles prepared from *Agaricus bisporus*. (ii) Analysis of the particles prepared from yeast.

TGA analysis showed that alginate sealed A3 particles are more thermo-stable with breakdown temperature around 290 °C than hollow β-glucan particles with breakdown temperature around 240 °C as shown in Fig. 9a and same in case of yeast derived particles, Y3 particles were found to have higher thermal stability with breakdown temperature around 450 °C than hollow β-glucan particles with breakdown temperature around 350 °C as shown in Fig. 9b. Overall, alginate sealed particles were having higher stability than the hollow β-glucan particles A1, and Y1.

#### Differential scanning calorimetry analysis (DSC)

**Figure 10:**
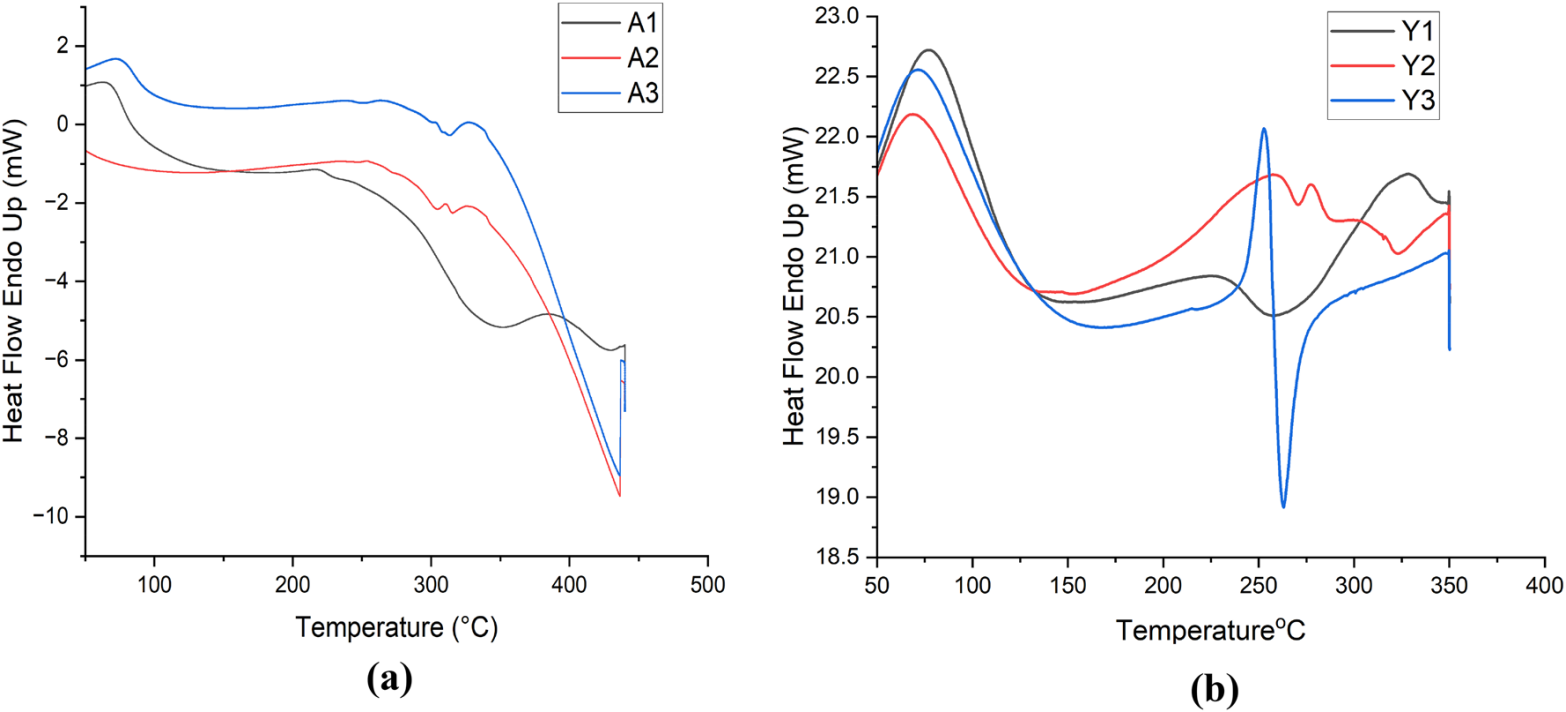
DSC analysis of the prepared particles (**a**) Analysis of the particles prepared from *Agaricus bisporus*. (**b**) Analysis of the particles prepared from yeast.

DSC analysis is indicative of higher stability with the increase in temperature in alginate sealed particles i.e., A3 and Y3 as shown in Fig. 10.

#### In vitro* drug release studies*

**Figure 11:**
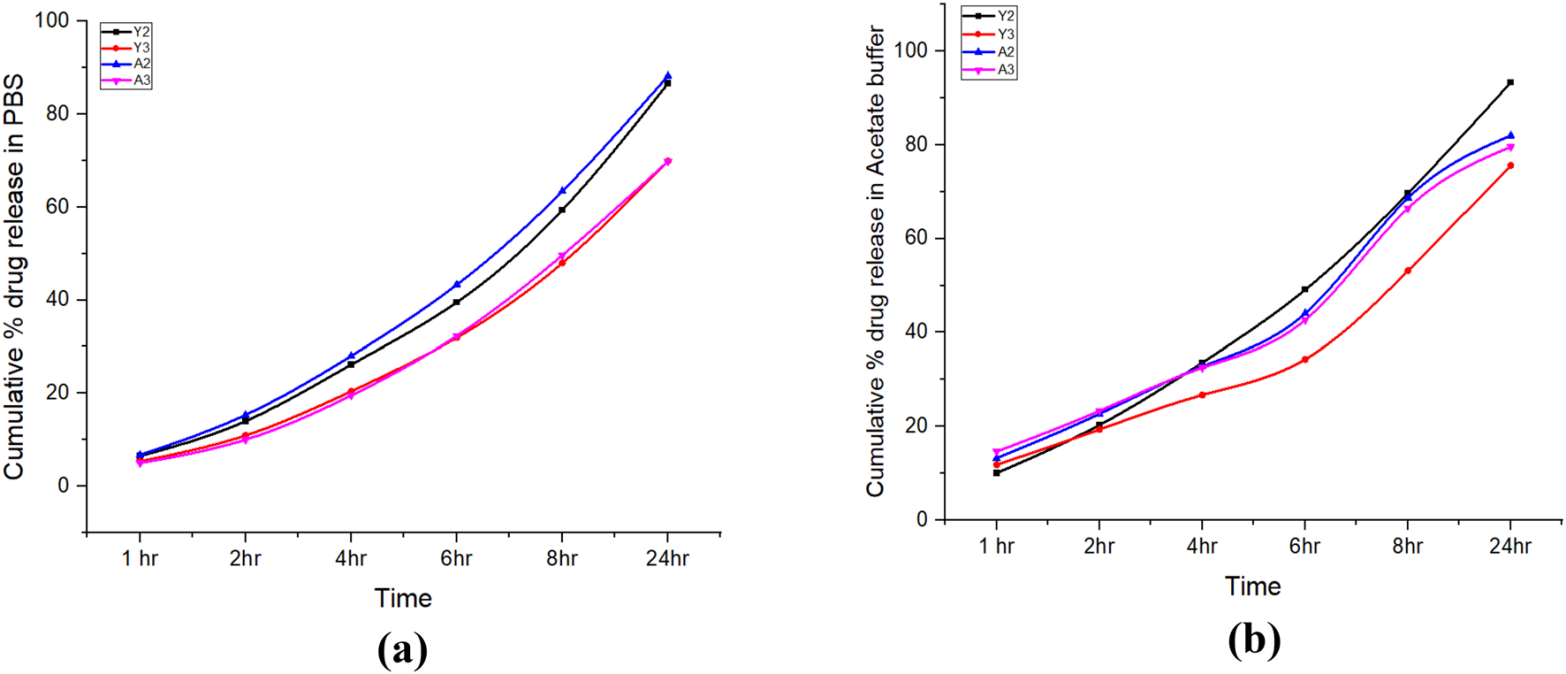
In vitro drug release studies showing slow and sustained release after the alginate coating. (**a**) Drug release in PBS (pH 7.4). (**b**) Drug release in Acetate buffer (pH 5.2).

Cumulative drug release in PBS (pH 7.4) for the A2 was 28% in 4 h and 88% in 24 h while A3 particles were showing 19% and 70% drug release in 4 and 24 h respectively indicating slow release of drug after alginate coating. Y2 release was found to be 26% in 4 h and 86% in 24 h while Y3 particles were showing 20% and 70% drug release in 4 and 24 h respectively as shown in Fig. 11a. In the acetate buffer (pH 5.2) drug release was 33% and 82% in 4 and 24 h respectively for the A2 and 32% and 79% for A3 in 4 and 24 h respectively. For the Y2 it was 33% and 93% in 4 h and 24 h respectively and 24% and 75% for the Y3 in 4 h and 24 h as shown in Fig. 11b. This study was clearly indicating that alginate coating is playing efficient role in the slow and sustained release of the Quercetin.

#### Determination of hemolytic inhibition activity

**Figure 12:**
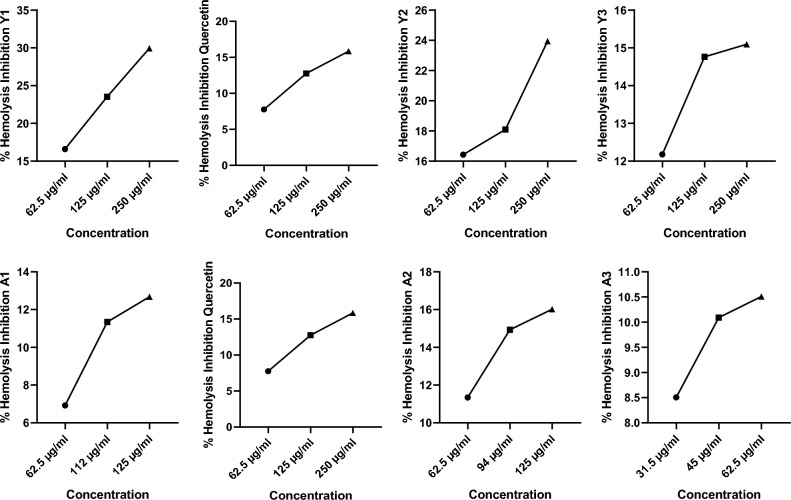
Hemolytic inhibition activity determination showing that alginate sealed particles was having less hemolytic inhibition activity.

% hemolytic inhibition was found to be 16, 23 and 29% for the Y1, 16, 18, 23% for the Y2 and 12, 14, 15% for the Y3 i.e. it was increasing with respect to the concentration of the compound but alginate sealed particles have less hemolytic inhibition activity. Quercetin was found to have 7, 12, and 15% while A1 have 6, 11, 12%, A2 have 11, 14, 16% and A3 have 8, 10, and 10% of hemolytic activity that was also increasing with the increasing concentration. Again, alginate sealed particles were having less hemolytic inhibition activity as shown in Fig. 12.

### Antioxidant activity determination

#### DPPH analysis showed increasing radical scavenging activity with concentration

**Figure 13:**
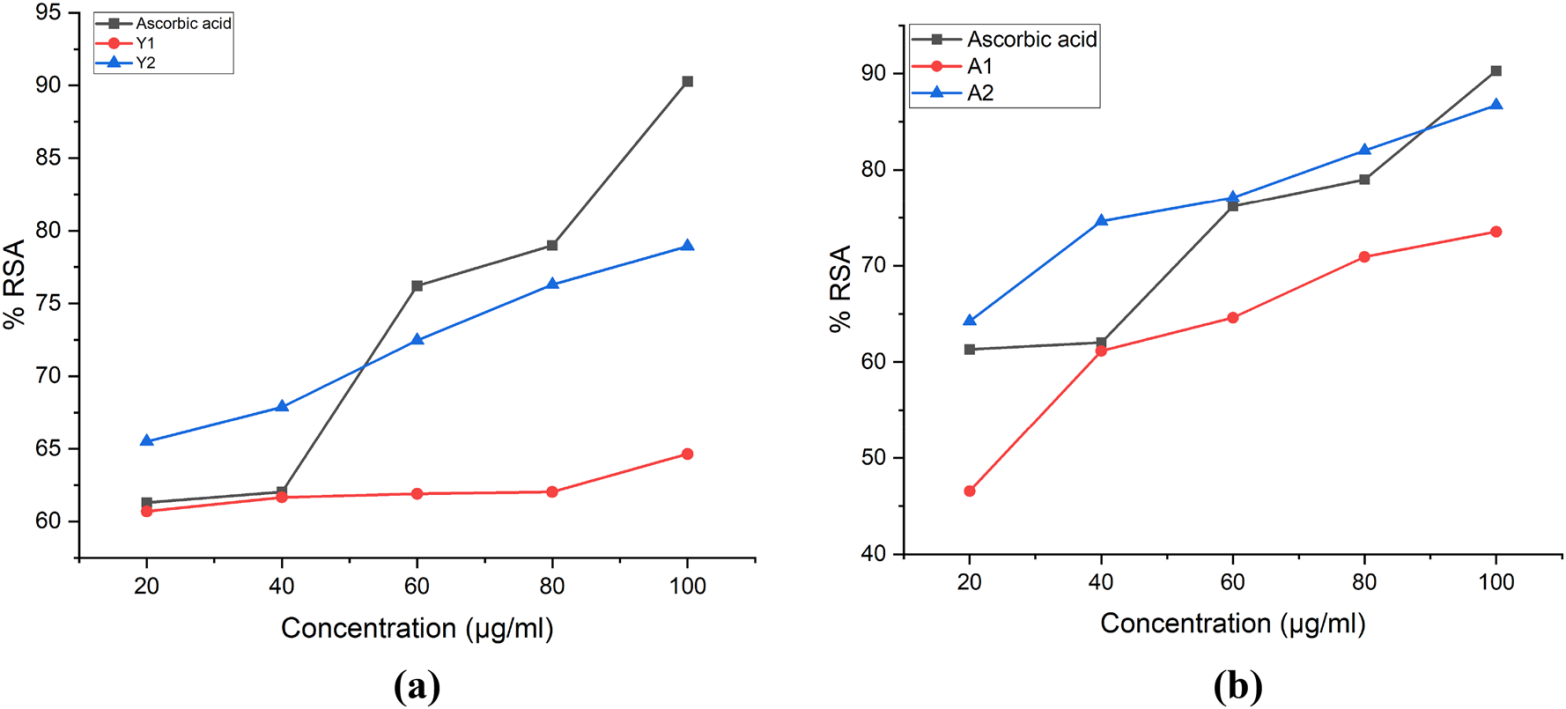
% RSA of the hollow β-glucan and Quercetin loaded β-glucan particles. (**a**) % RSA of particles derived from *Agaricus bisporus* (**b**) % RSA of particles derived from yeast.

In the DPPH radical scavenging activity determination, we found that Quercetin-loaded particles were having more RSA than hollow β-glucan particles as shown in Fig. 13. Maximum % RSA was reported at 100 µg/ml of the samples.

#### Reducing power assay

**Figure 14:**
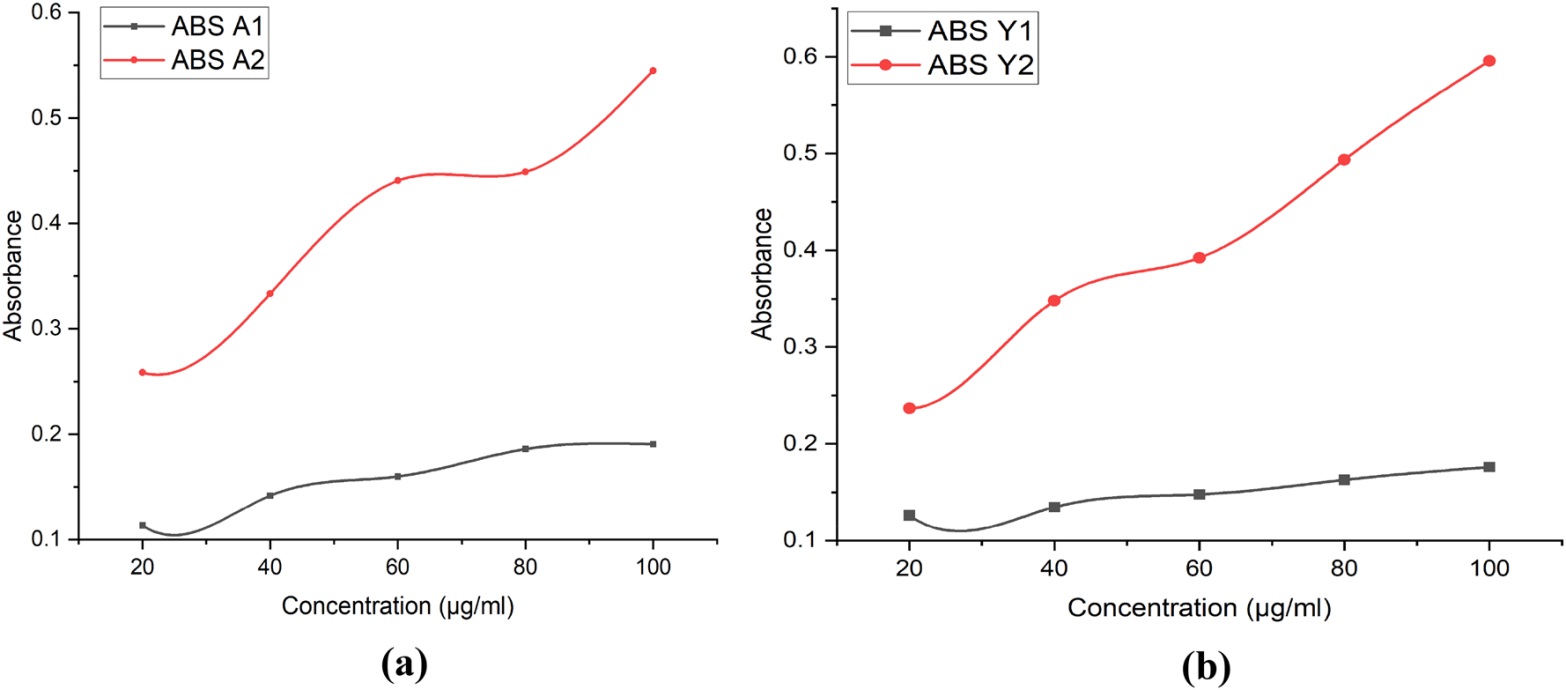
Reducing power of the hollow β-glucan and Quercetin loaded β-glucan particles. (**a**) Reducing power of particles derived from *Agaricus bisporus* (**b**) Reducing power of particles derived from yeast.

Reducing power estimation was carried out with the help of measuring the absorbance. Absorbance of the samples was found to increase with concentration of the particles indicating dose dependent increase of the reducing power. Quercetin loaded β-glucan particles were having more reducing power than the hollow β-glucan particles as shown in Fig. 14.

#### Determination of the total Phenolic content (TPC)

**Figure 15:**
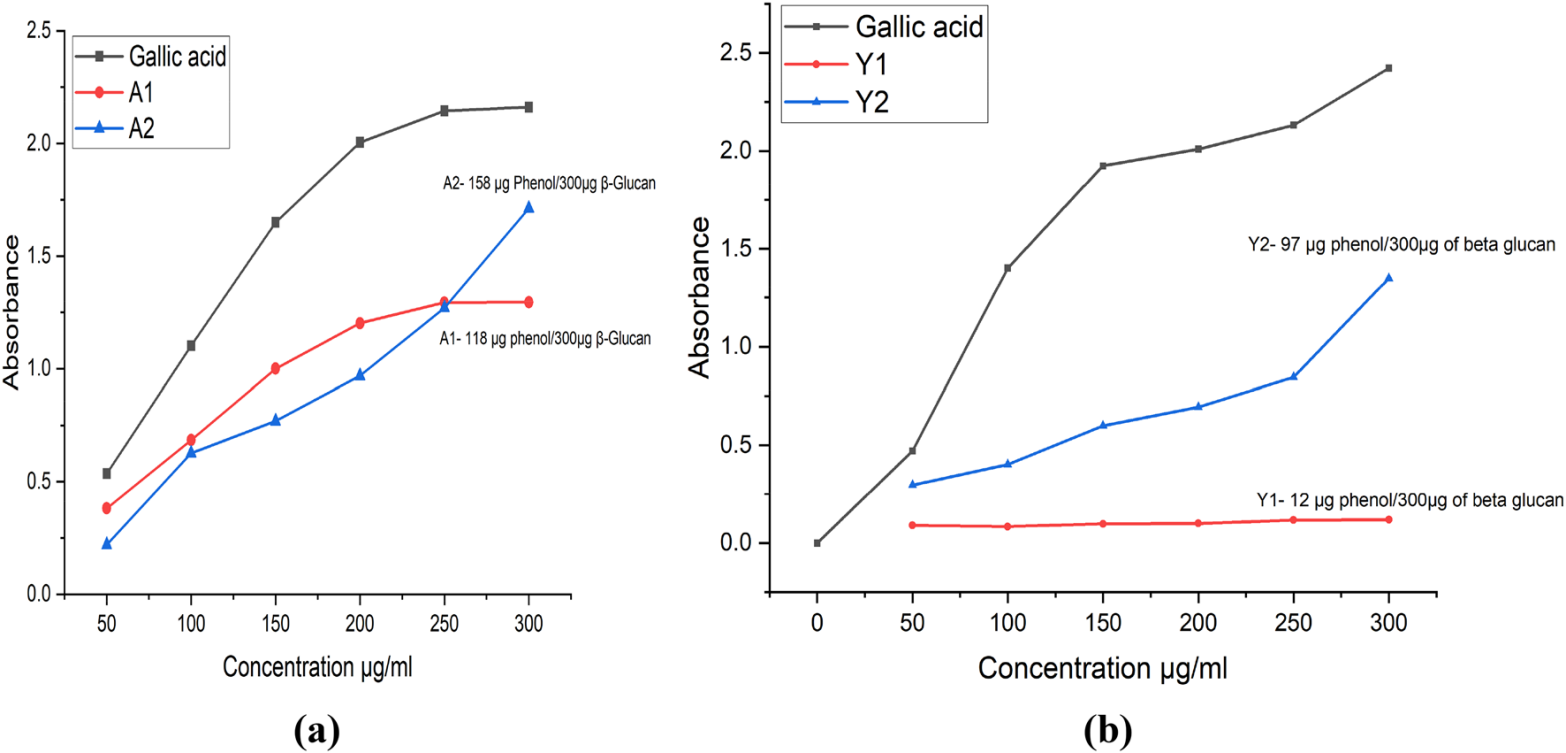
Total phenolic content of the hollow β-glucan and Quercetin loaded β-glucan particles. (**a**) TPC of particles derived from *Agaricus bisporus*. A1 is having 118 µg of phenol/300 µg of the β-glucan while A2 was having 158 µg of phenol/300 µg of the Quercetin loaded β-glucan (**b**) Reducing power of particles derived from yeast. Y1 is having 12 µg of phenol/300 µg of the β-glucan while Y2 was having 97 µg of phenol/300 µg of the Quercetin loaded β-glucan particles.

Phenolic content of the compound is directly proportional to their antioxidant activity^51^. Quercetin is considered as good antioxidant compound. In our study, we found that Phenolic content of the Quercetin-loaded particles was more than hollow particles as shown in Fig. 15. Comparison of antioxidant activities of particles is shown in Table 2.

**Table 2:** Summary of MTT, drug release, antioxidant and anticancerous activity.

S. No	Particle	IC50 (MTT)	Size Analysis	Antioxidant Activity			In vitro drug release	
				% RSA	Reducing power	Total Phenolic content	PBS (pH 7.4)	Acetate buffer (pH 5.2)
1	A1	112 µg/ml	119.8 nm	73%	0.190	120 μg phenol/300 μg β-Glucan	–	–
2	A2	94 µg/ml	132.8 nm	86%	0.545	150 μg Phenol/300 μg β-Glucan + Q	88%	32%
3	A3	45 µg/ml	214.1 nm	–	–	–	70%	79%
4	Y1	175 µg/ml	161.9 nm	64%	0.176	10 μg phenol/300 μg of beta glucan	–	–
5	Y2	200 µg/ml	113.5 nm	77%	0.596	96 μg phenol/300 μg of β-Glucan + Q	86%	93%
6	Y3	150 µg/ml	156.9 nm	–	–	–	70%	75%

### Determination of anticancer activity

#### Assessment of the cell viability with MTT assay

**Figure 16:**
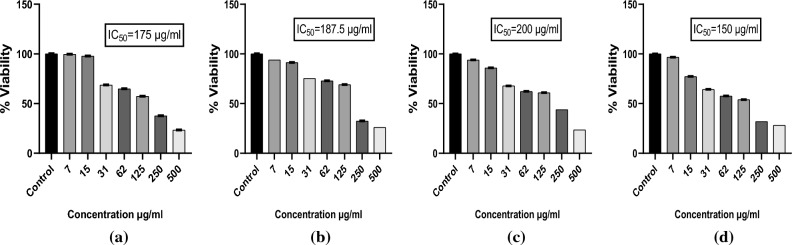
MTT of particles derived from yeast β-glucan. (**a**) Cell viability of PC3 after treatment with Y1 (**b**) Cell viability of PC3 after treatment with Quercetin (**c**) Cell viability of PC3 after treatment with Y2 (**d**) Cell viability of PC3 after treatment with Y3.

**Figure 17:**
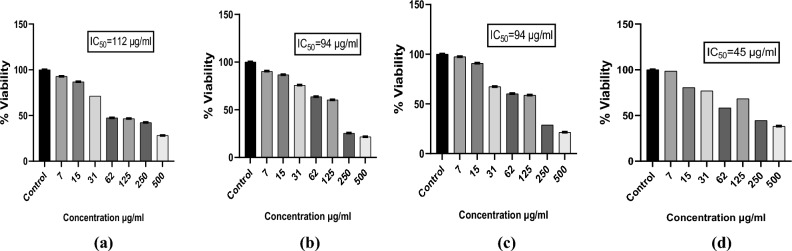
MTT of the particles derived from *Agaricus bisporus* β-glucan. (**a**) Cell viability of PC3 after treatment with A1 (**b**) Cell viability of PC3 after treatment with Quercetin (**c**) Cell viability of PC3 after treatment with A2 (**d**) Cell viability of PC3 after treatment with A3.

Cell viability of the prepared particles and Quercetin was assessed using MTT assay. There was a dose dependent decrease of the viability of PC3 cells with IC_50_ 175, 187.5, 200, 150 µg/ml for the Y1, Quercetin, Y2 and Y3 as shown in Fig. 16. Same trend was observed for the viability of the PC3 cells with the IC_50_ 112, 94, 94, 45 µg/ml for the A1, Quercetin, A2 and A3 as shown in Fig. 17. Less IC_50_ in *Agaricus bisporus* derived particles indicating the efficient anticancer activity of these particles. Concentrations below and above the IC_50_ and IC_50_ were taken for further evaluations.

#### Morphological observations

**Figure 18:**
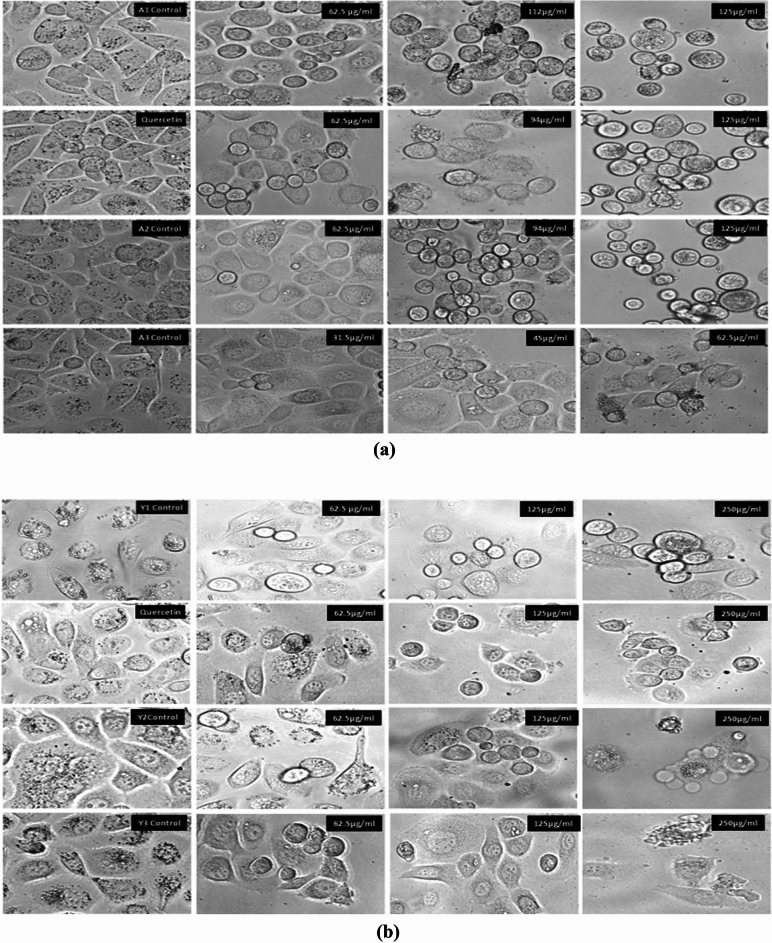
Morphological changes in the PC3 cells as observed with the microscope. (**a**) PC3 cells after treatment with *Agaricus bisporus* derived particles. At the highest concentration, cells showed maximum morphological changes. (**b**) PC3 cells after treatment with yeast derived particles.

Clear morphological changes were observed with the help of FLoid Imaging microscope after treatment of the PC3 cells with different doses of all the particles. At the highest concentration, cells have lost their actual shape and appeared almost round in shape as shown in Fig. 18. Alginate sealed particles were having visible ruptured cells.

#### Reactive oxygen species (ROS) generation

**Figure 19:**
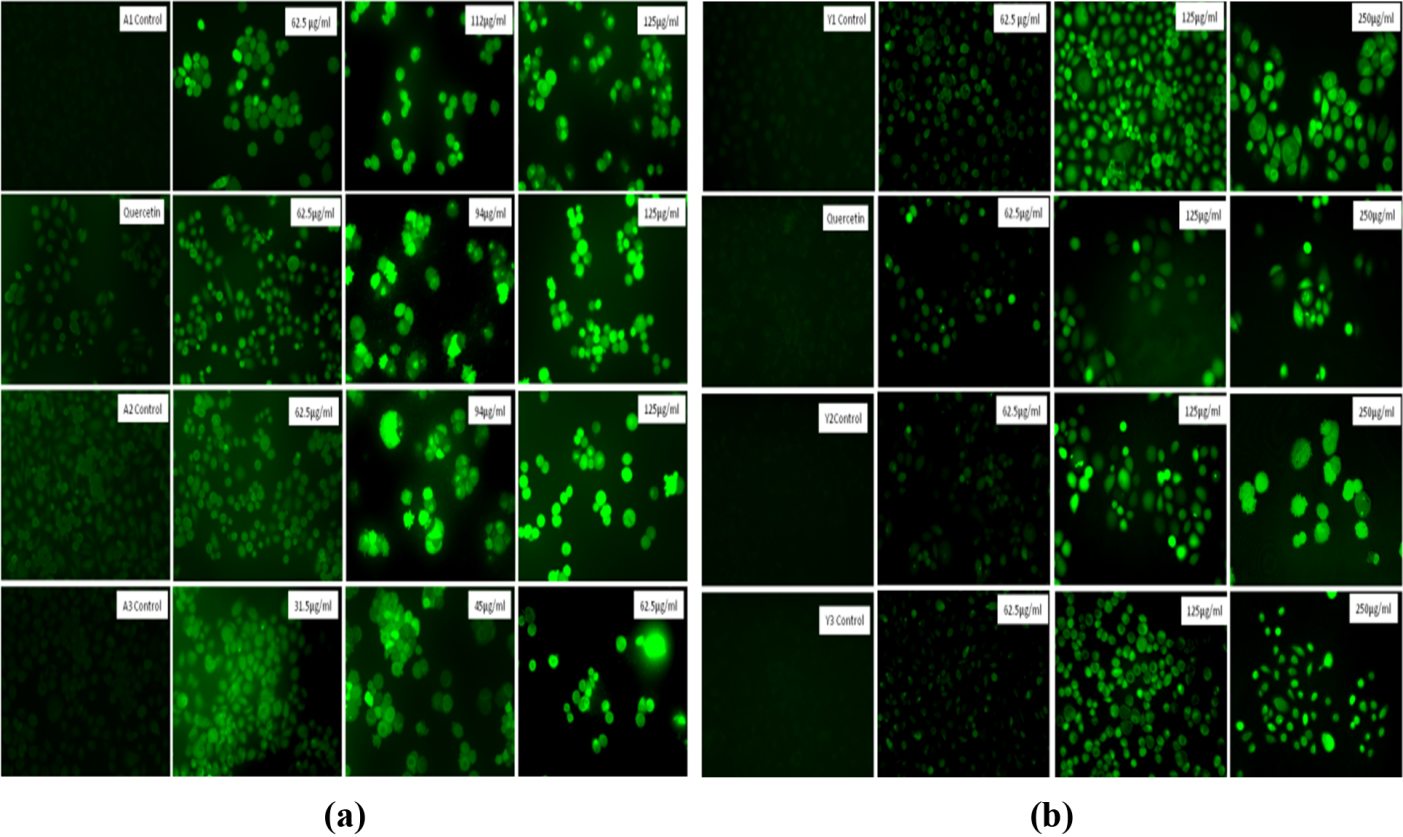
ROS generation in the PC3 cells as observed with the microscope. ROS generation was increased in a dose dependent manner. (**a**) PC3 cells after treatment with *Agaricus bisporus* derived particles. (**b**) PC3 cells after treatment with yeast derived particles.

ROS generation was observed to increase in PC3 cells in a dose dependent manner. Excessive ROS generation can lead to oxidative damages leading to the apoptosis of the cancer cells^52^. Fluorescence intensity increased and was maximum at the highest concentration indicating maximum ROS generation at the highest concentration that can lead to cell death as shown in Fig. 19.

#### Determination of nuclear morphology and DNA fragmentation with DAPI staining

**Figure 20:**
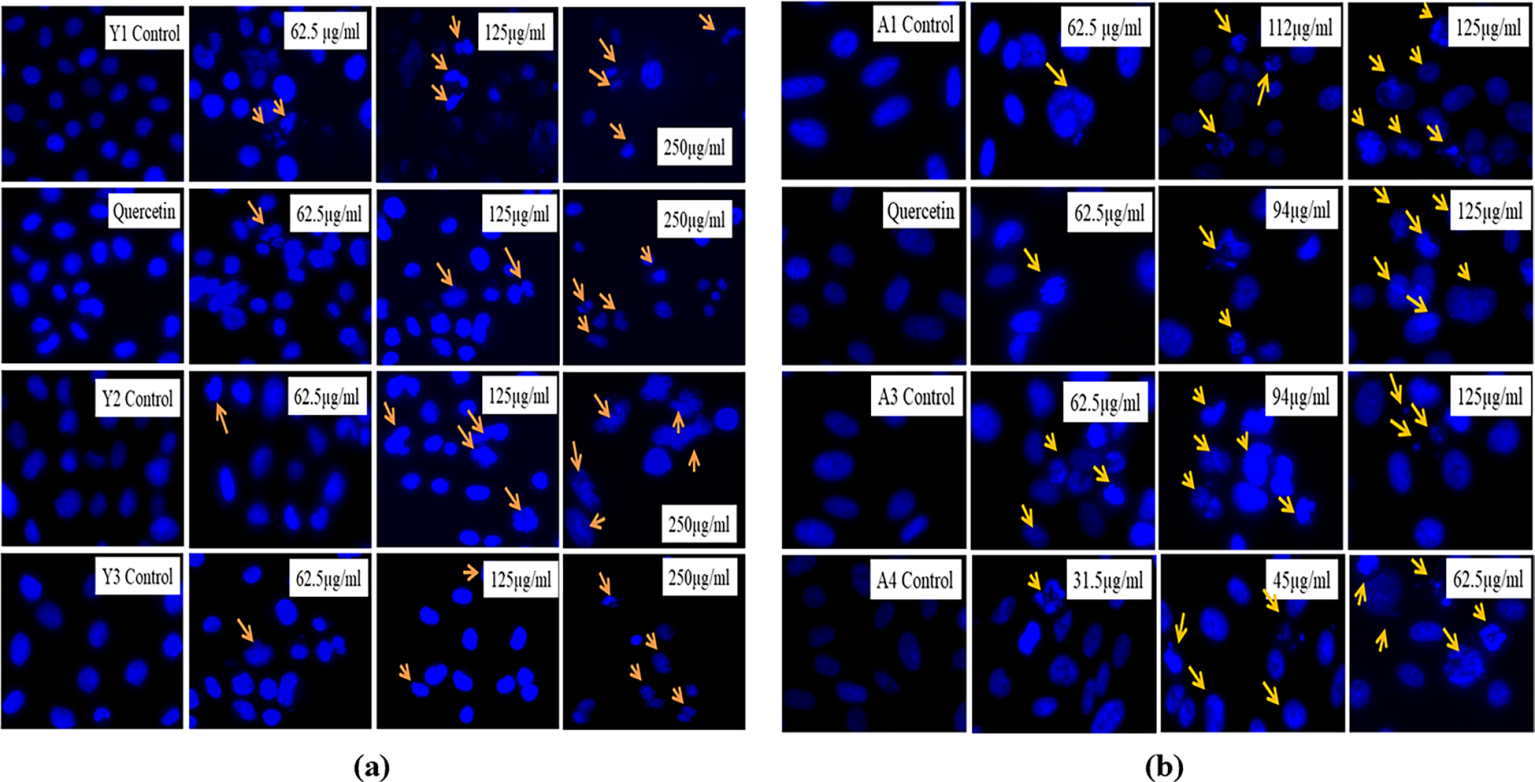
Nuclear morphology changes and DNA fragmentation in the PC3 cells as observed with the microscope. (**a**) PC3 cells after treatment with *Agaricus bisporus* derived particles. (**b**) PC3 cells after treatment with yeast derived particles. Condensation of the nuclear material and DNA fragmentation is shown with the help of arrows.

The effect of particles on the PC3 cells was assessed with DAPI staining that is specific for the binding with nucleus. Clear nuclear morphological changes can be seen in Fig. 20. We found that nuclear deformities were increasing with the increase in the treatment concentration of the particles.

#### Apoptosis detection with Propidium iodide (PI) staining

**Figure 21:**
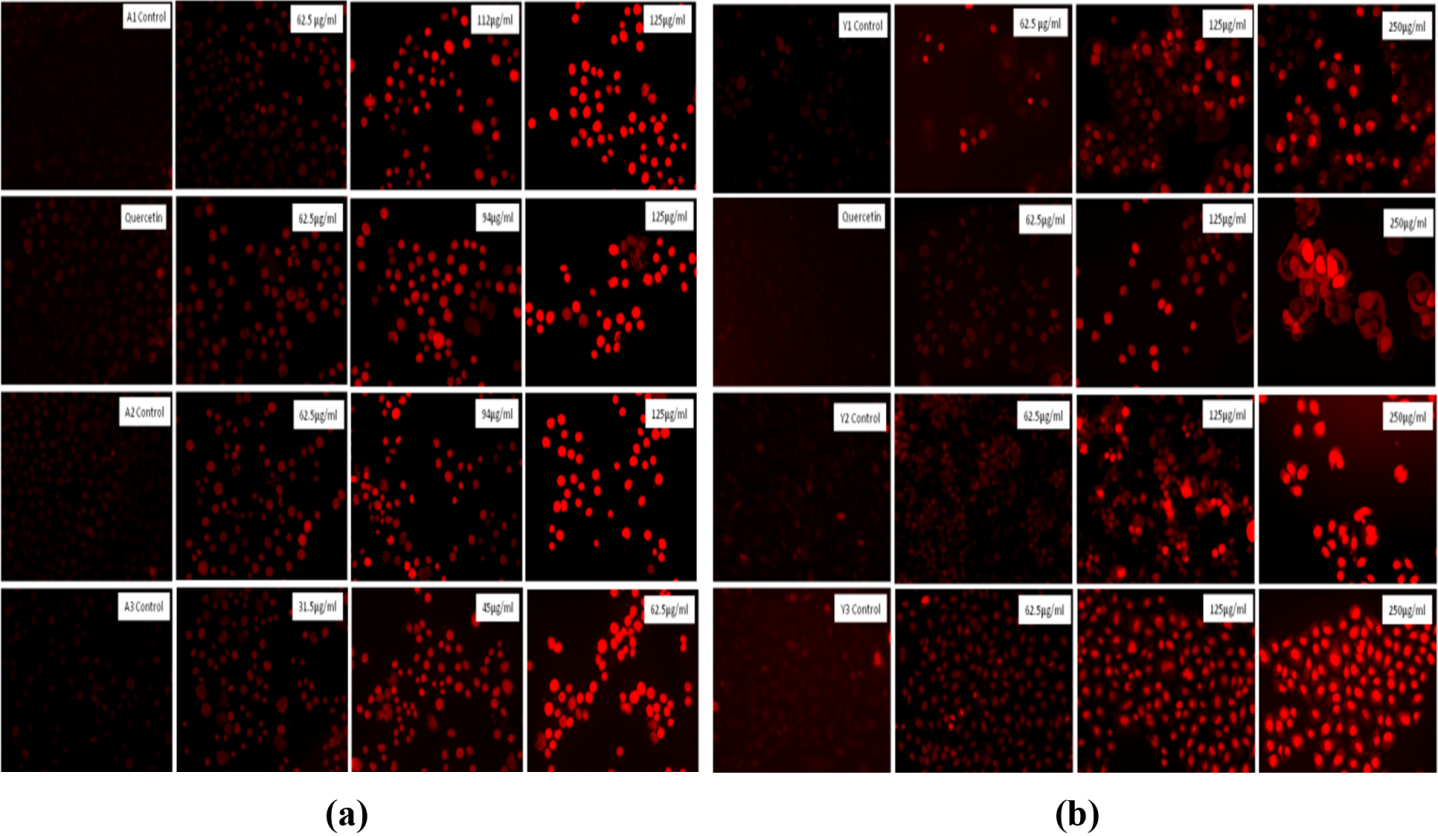
Apoptosis determination in the PC3 cells as observed with the fluorescence microscope. (**a**) PC3 cells after treatment with *Agaricus bisporus* derived particles. (**b**) PC3 cells after treatment with yeast derived particles.

PI is a very specific dye that only binds with the dying or apoptotic cells resulting in red color fluorescence^53^. It is not able to permeate the cell membrane of the healthy cells. In our study we found the increase in fluorescence intensity of PI in a dose dependent manner as shown in Fig. 21. In control cells, there was no or minimum fluorescence showing that cell membrane in integrate in those cells while a continuous increase in fluorescence was confirming that cells are not able to maintain the membrane integrity that can ultimately lead to the cell death.

#### Acidic organelles activity analysis through LysoTracker Red DND-99

**Figure 22:**
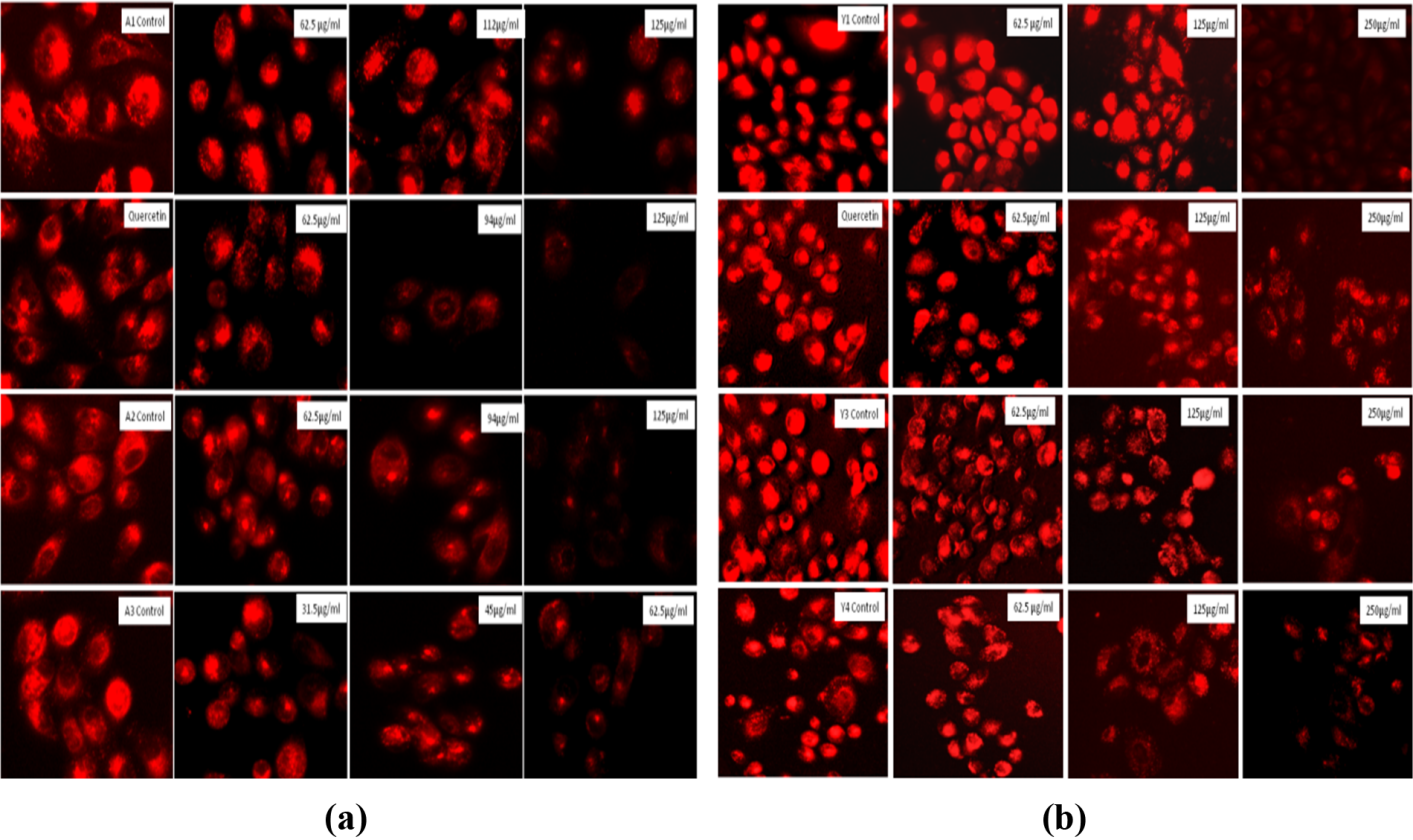
LysoTracker staining in the PC3 cells as observed with the fluorescence microscope for the determination of acidic organelles. (**a**) PC3 cells after treatment with *Agaricus bisporus* derived particles. (**b**) PC3 cells after treatment with yeast derived particles.

LysoTracker Red DND-99 dye is specific for the accumulation in acidic organelles due to their acidic pH^54^. Due to their accumulation, they show intense red florescence in the untreated cells but this decreases due to the change in pH of the apoptotic cells. We found a dose dependent decrease in florescence intensity indicating the pH alteration that can result in the apoptosis of the cells as shown in Fig. 22.

#### Altered mitochondrial membrane potential observed through MitoTracker red CMX-ROS

**Figure 23:**
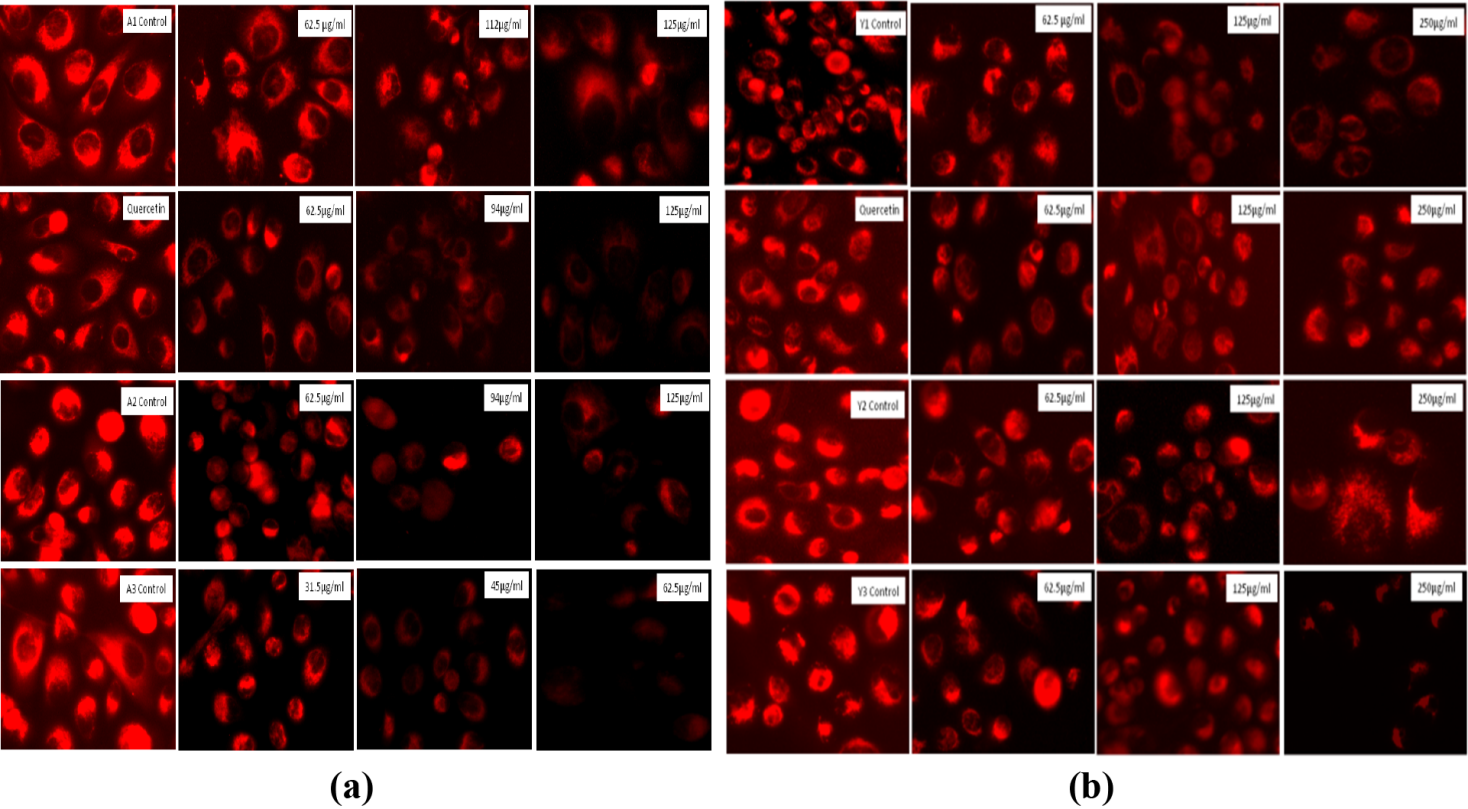
MitoTracker Red staining in the PC3 cells as observed with the fluorescence microscope for the determination of mitochondrial activity. (**a**) PC3 cells after treatment with *Agaricus bisporus* derived particles. (**b**) PC3 cells after treatment with yeast derived particles. A continuous decrease in fluorescence was observed in both of the cases.

Mitochondria have negative membrane potential for its normal activity^55^. MitoTracker Red CMX-ROS is a cationic dye that enters in the healthy cells due to its negative membrane potential and accumulates there due to complete integrity of the cell membrane resulting in intense red fluorescence. Due to the alteration in the membrane potential (shift to positive), mitochondria are not able to retain this cationic dye and start losing their fluorescence. We found a dose dependent decrease in fluorescence of MitoTracker dye stained cells indicating the membrane potential alteration that can lead to the cell death as shown in Fig. 23.

#### Early and late apoptosis detection with acridine orange and ethidium bromide (AO/EtBr) staining

**Figure 24:**
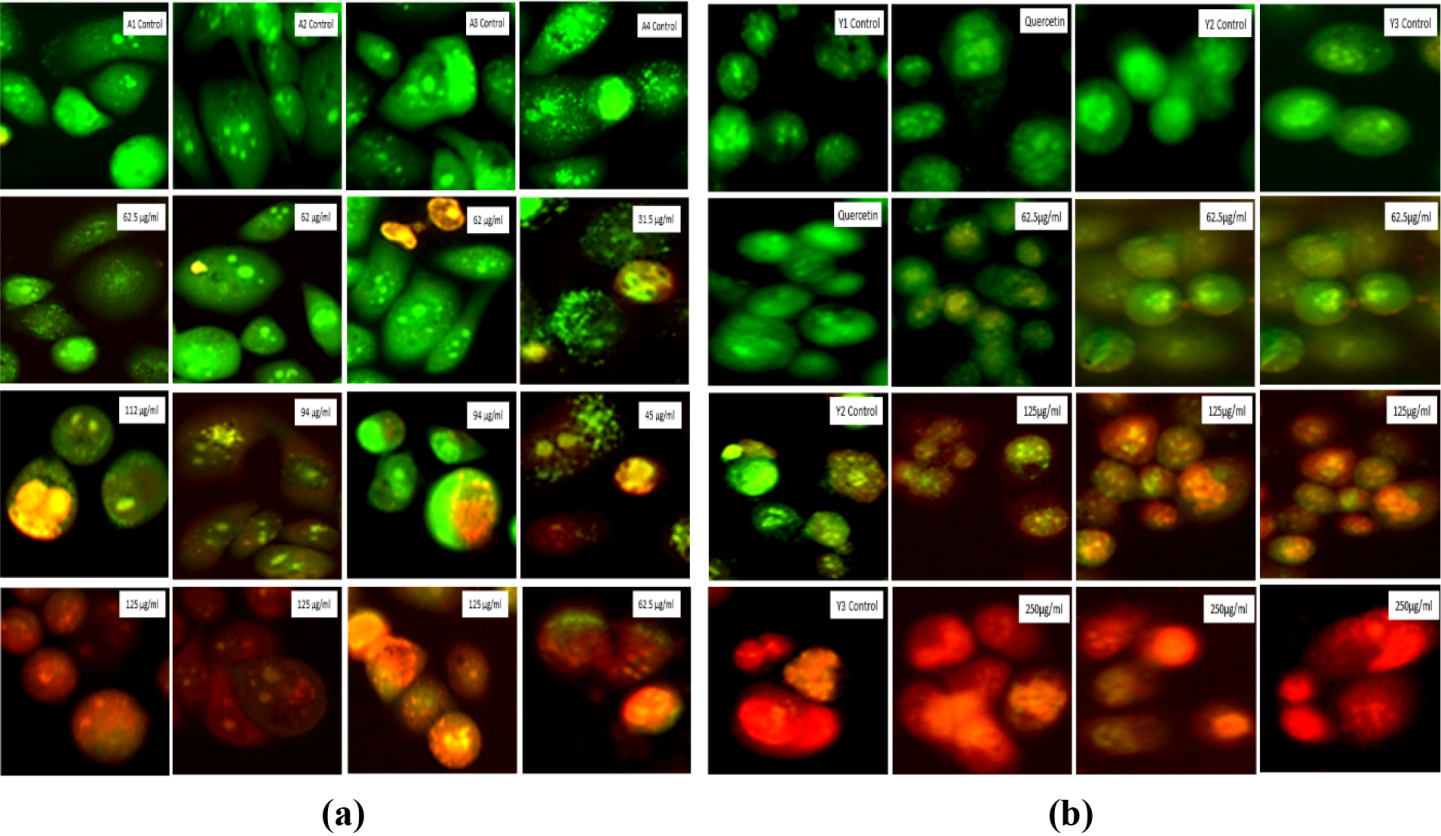
Dual staining of the PC3 cells showing cells in early and late apoptotic stage. (**a**) PC3 cells after treatment with *Agaricus bisporus* derived particles. (**b**) PC3 cells after treatment with yeast derived particles.

AO/EtBr dual staining is considered very efficient method for the detection of early and late apoptotic cells. AO dye is permeable to all the cells and gives florescence in green color while EtBr is only permeable to the cells undergoing apoptosis and gives red fluorescence. In the dual staining, cells in early apoptotic stage show yellowish-orange color while cells in late apoptotic stage show orange-red color. We have found a continuous decrease in green fluorescence in a dose dependent manner where some of the cells appeared as yellow-orange-red color showing cells in early and late apoptosis as shown in Fig. 24.

#### Wound healing/ cells scratch assay

**Figure 25:**
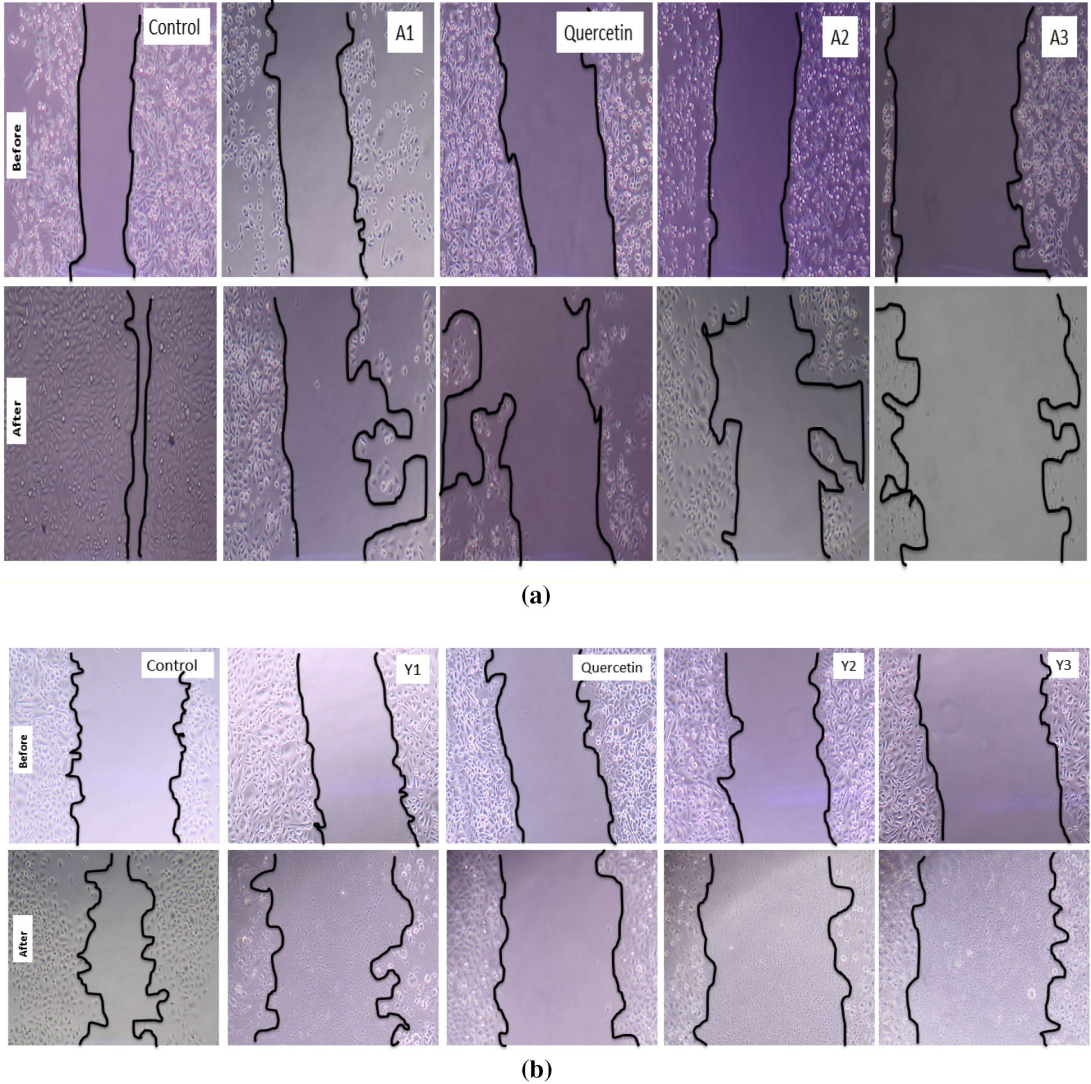
Cells Scratch assay showing the migration of cells in the control wells is more than the migration in the treated wells and cells were able to grow in control wells thereby covering almost area of the scratch while treated wells were not able to cover the area and there was an increase in the scratch area.

Area of the wound in the treated wells was increased by around 30% for the quercetin loaded particle and around 40% in case of alginate sealed particles for particles derived from *Agaricus bisporus*. In articles derived from yeast, cells scratch area of quercetin loaded particles was increased upto 47% while in case of alginate sealed particles, it increased up to 13% indicating the inhibition of the cell migration by the treatment with prepared particles. Maximum migration was found in control wells and area reduced to 50–25% after 24 h as shown in Fig. 25.

#### DNA fragmentation assay

**Figure 26:**
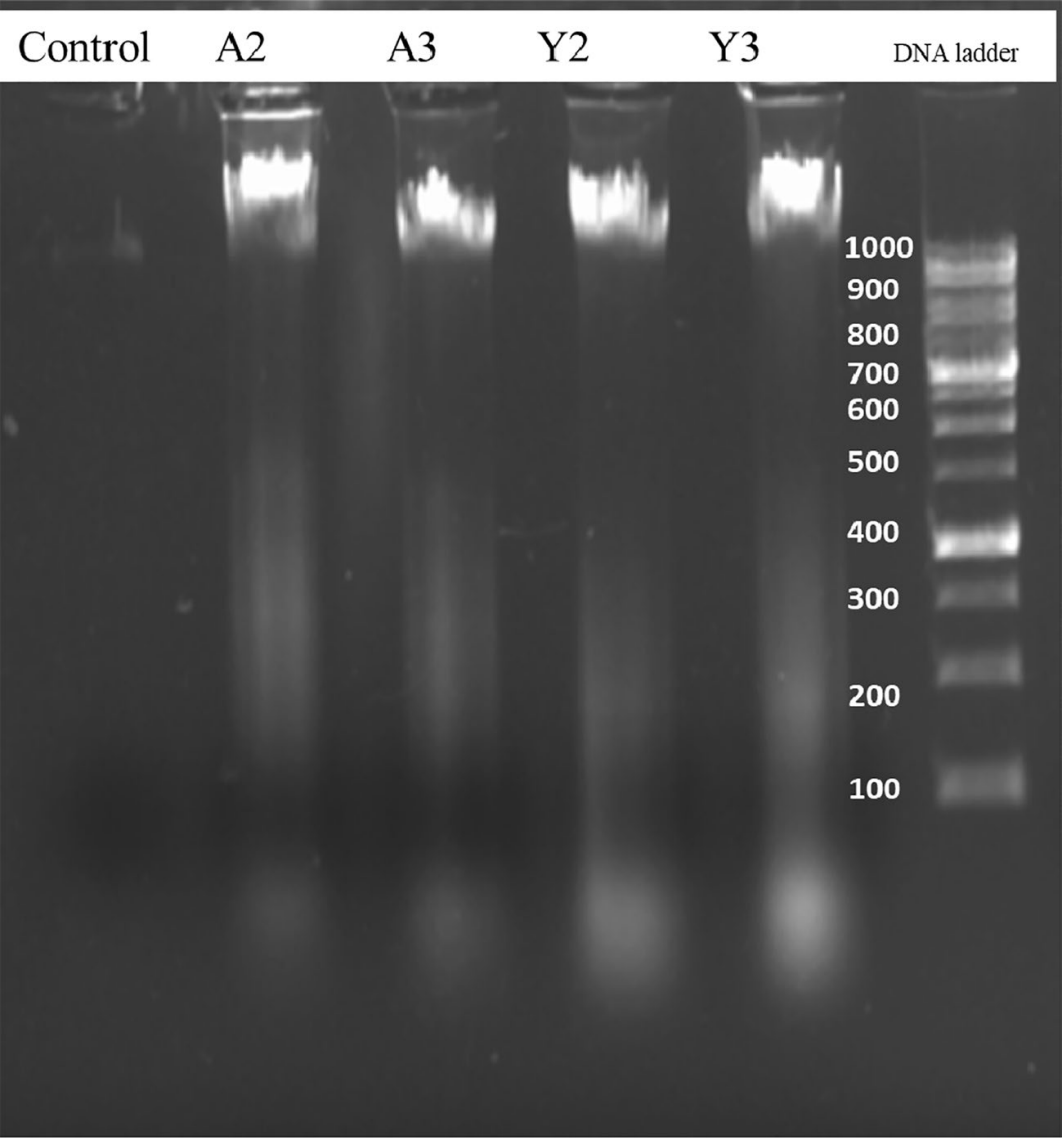
DNA fragmentation assay showing the smears of the extracted DNA after treatment indicating that DNA is being fragmented due to the treatment of the particles.

Oligonucleosomal DNA degradation indicates the late stage of apoptosis and DNA appears as smear due to the necrosis of the cells that results from the random degradation of the cells^56^. We also found smears of the extracted DNA for all the DNA of treated cells as shown in Fig. 26.

## Discussion

β-glucan prepared from the yeast was white in color while β-glucan prepared from *Agaricus bisporus* was brown in color. As mushrooms are rich in the pigment melanin so that might be responsible for the brown color of the β-glucan obtained from the *Agaricus bisporus*. Quantification of β-glucan through aniline blue and Congo red staining confirmed the single and triple helical conformation of the β-glucan. In the aniline blue staining, NaOH removes the 1, 6 branching of the β-glucan thereby resulting in the un-branched β-1,3 linkage containing structure. This un-branched structure gives fluorescence upon reaction with aniline blue. The same pattern of the increasing RFU was also observed by^22^. Confirmation of triple helical structure of β-glucan is crucial as it is involved in the functional activities and binding of β-glucan with receptors^57^. Congo red dye forms complex only with tridimensional structure of the polysaccharide β-glucan and this was also reported by a researcher for the shiitake mushrooms where only fraction with the (1 → 6)-β-D-glucan and (1 → 3)-(1 → 6)-β-D-glucan showed the bathochromic shift^58^. We have performed size analysis using zeta-sizer and SEM both. As zeta sizer works on the principle of dynamic light scattering and it has low size resolution ability as it suppresses the small particles in size distribution. As it is not able to measure the particle size in multimodal distribution, we have performed SEM to get the actual size and morphology^59^. We got high sizing resolution and accuracy with the SEM analysis along with higher size than the zeta sizer. This might be due to the particle aggregation during the process of drying when preparing sample for the SEM. PDI value of 0.3 or below is considered good and indicates a homogenous particle population. In our study PDI value for all the particles were between 0.2 and 0.3 showing homogeneity of the particles^60^. In a study, peak value at 3335.96 showed the OH stretch of the Quercetin in the Quercetin Loaded Polymeric Mixed Micelles^61^. Alginate sealing leads to a negative zeta potential of the prepared particles that was also reported by^62^ in the coating of chitosan nanoparticles with sodium alginate.

β-glucan peaks were observed with FTIR analysis confirmed that extracted particles are β-glucan and obtained spectra was in the range of various previously reported studies^47^. In the Quercetin loaded and alginate sealed particles, exact spectra are not reported but we found the major peaks of Quercetin and Sodium alginate in our prepared particles that is a clear indication that particles have Quercetin loading and alginate sealing. SEM and XRD analysis confirmed morphology and crystalline nature of the prepared particles while TGA and DSC represented the stability of the particles in which we have found that alginate sealed particles prepared from *Agaricus bisporus* and yeast are having more stability than the hollow particles. Various studies reported the higher stability of quercetin loaded formulation on various temperatures. In a study, researcher obtained the stability of quercetin loaded system at 4 °C, 25 °C, and 37 °C. System was able to maintain its encapsulation efficiency up to 90% for 9 months^63^. In another study, physiochemical properties of quercetin loaded polymeric micelles were assessed after 6-month storage and 5% mannitol were used as lyoprotectant. Researchers found that freeze dried quercetin loaded polymeric micelles were stable with little or no change in particle size, PDI value, and encapsulation efficiency along with no any shrinkage or collapse of the dried particles^64^. Furthermore, research investigated the quercetin-loaded nano-emulsions on 21 and 37 °C temperature after the storage of 3 months. Findings indicated that there was an increase in size and PDI value after 3 months. This study was also supported by another study that state that long term storage at temperatures above 55 °C leads to instability of quercetin loaded nano-emulsion than the storage at lower temperatures (21 °C)^65^.

Sodium alginate is already explored by many researchers as a coating material for the slow and sustained release of the drug from compound^66^. With the help of in vitro drug release assay, we found that alginate coating is slowing down the release of the Quercetin in the release media. We also reported higher slow release in the PBS at pH 7.4 than the acetate buffer at pH 5.2. This might be due to the fact that β-glucan binding receptors are present in the intestine that have a pH value around 7.4^67^. Hemolytic inhibition activity was low for the alginate sealed particles in our study that is representing the main characteristics of the alginate i.e. biocompatibility, and low toxicity^68^. In our study, we reported a very efficient antioxidant activity of the Quercetin loaded β-glucan particles than the hollow β-glucan particles. Quercetin is a flavonoid compound that is reported to have a very good antioxidant activity^69^.

Moreover, we have assessed the anticancer activity of all the prepared particles and found that the alginate sealed particles *i.e.* A3 and Y3 are having lowest IC_50_ values so they can be considered as good anticancer agent against prostate cancer. ROS generation was found to increase in a dose dependent manner that was indicating the apoptosis of the cancer cells due to the oxidative stress. Quercetin is reported to cause cell death in cancer cells due to the generation of ROS in many studies^70^ and slow and sustained release of the Quercetin from the hollow β-glucan particles due to alginate sealing will surely enhance its potential towards efficient anticancer activity. Cellular apoptosis was also confirmed with the various organelle specific dye staining such as DAPI, MitoTracker and LysoTracker that were also indicating the apoptotic cell death due to the treatment with the particles. AO/EtBr staining was found to show cells in the early and late apoptotic stage and cell scratch assay confirmed that cells are losing their capability to migrate. Similar pattern was observed in a study in which Quercetin was effectively found to inhibit the migration of the ovarian cancer cells^71^. This can be very beneficial because migration of cells from a place to another can result in the metastasis of the prostate cancer. DNA fragmentation results were also showing that cell’s DNA is getting disintegrated due to treatment with the prepared particles. DNA fragmentation-mediated apoptosis response of hexagonal ZnO particles was reported in a study against human prostate cancer cells^72^. Overall studies suggest that Quercetin loading and alginate sealing has improved the anticancer activity of the β-glucan particles.

## Conclusion

β-glucan and Quercetin are reported to have anticancer activity in many studies specially Quercetin is found to show efficient anticancer activity against prostate cancer. In conclusion, we can say that loading of the Quercetin in β-glucan leads to its targeted delivery and alginate sealing will impact its overall slow and sustained release for the treatment of the prostate cancer. Quercetin-loaded particles were also found to have good antioxidant activity that can contribute to its anticancer activity by activating various oxidative enzymes. Although, we reported a good anticancer activity of quercetin-loaded and alginate-sealed particles but some other studies such as in vivo and clinical trials can be performed to confirm and validate our findings.

## Supplementary Information

Supplementary Information 1.

Supplementary Information 2.

## Data Availability

All the experimental data given in the manuscript and raw data of the results will be available on request. Our manuscript does not comprise of western blot and no need to provide raw blot images. Rashmi Trivedi should be contacted if someone wants to request the data from this study.

## References

[1] Mirzaei, S. *et al.* Molecular landscape of LncRNAs in prostate cancer: A focus on pathways and therapeutic targets for intervention. *J. Exp. Clin. Cancer Res.* **41**, 214. 10.1186/s13046-022-02406-1 (2022). 35773731 10.1186/s13046-022-02406-1 PMC9248128

[2] Abeshouse, A. *et al.* The molecular taxonomy of primary prostate cancer. *Cell.* **163**, 1011–1025. 10.1016/j.cell.2015.10.025 (2015). 26544944 10.1016/j.cell.2015.10.025 PMC4695400

[3] Abida, W. *et al.* Prospective genomic profiling of prostate cancer across disease states reveals germline and somatic alterations that may affect clinical decision making. *JCO Precis. Oncol.* **1**, 1–16. 10.1200/po.17.00029 (2017). 10.1200/PO.17.00029 PMC5558263 28825054

[4] Kasivisvanathan, V. *et al.* MRI-targeted or standard biopsy for prostate-cancer diagnosis. *N Engl J Med.* **378**, 1767–1777. 10.1056/nejmoa1801993 (2018). 29552975 10.1056/NEJMoa1801993 PMC9084630

[5] Akramienė, D., Kondrotas, A., Didžiapetrienė, J. & Kėvelaitis, E. Effects of β-glucans on the immune system. *Medicina.* **43**, 597. 10.3390/medicina43080076 (2007). 17895634

[6] Volman, J. J., Ramakers, J. D. & Plat, J. Dietary modulation of immune function by β-glucans. *Physiol, Behav.* **94**, 276–284. 10.1016/j.physbeh.2007.11.045 (2008). 18222501 10.1016/j.physbeh.2007.11.045

[7] Yu, J. *et al.* Effects of oat beta-glucan intake on lipid profiles in hypercholesterolemic adults: A systematic review and meta-analysis of randomized controlled trials. *Nutrients.* **14**, 2043. 10.3390/nu14102043 (2022). 35631184 10.3390/nu14102043 PMC9147392

[8] Salehi, B. *et al.* Therapeutic potential of quercetin: New insights and perspectives for human health. *Acs Omega.* **5**, 11849–11872. 10.1021/acsomega.0c01818 (2020). 32478277 10.1021/acsomega.0c01818 PMC7254783

[9] Gasmi, A. *et al.* Quercetin in the prevention and treatment of coronavirus infections: a focus on SARS-CoV-2. *Pharmaceuticals.* **15**, 1049. 10.3390/ph15091049 (2022). 36145270 10.3390/ph15091049 PMC9504481

[10] Lotfi, N. *et al.* The potential anti-cancer effects of quercetin on blood, prostate and lung cancers: An update. *Frontiers in immunology* **14**, 1077531. 10.3389/fimmu.2023.1077531 (2023). 36926328 10.3389/fimmu.2023.1077531 PMC10011078

[11] Essa, D., Kondiah, P. P., Kumar, P. & Choonara, Y. E. Design of chitosan-coated, quercetin-loaded PLGA nanoparticles for enhanced PSMA-specific activity on LNCap prostate cancer cells. *Biomedicines* **11**(4), 1201. 10.3390/biomedicines11041201 (2023). 37189819 10.3390/biomedicines11041201 PMC10136298

[12] Hussain, Y. *et al.* Quercetin and its nano-scale delivery systems in prostate cancer therapy: paving the way for cancer elimination and reversing chemoresistance. *Cancers* **13**(7), 1602. 10.3390/cancers13071602 (2021). 33807174 10.3390/cancers13071602 PMC8036441

[13] Liu, J. *et al.* Preparation, characterization, stability, and controlled release of chitosan-coated zein/shellac nanoparticles for the delivery of Quercetin. *Food Chem.*10.1016/j.foodchem.2024.138634 (2024). 38330608 10.1016/j.foodchem.2024.138634

[14] Jakobek, L. *et al.* Adsorption between quercetin derivatives and β-glucan studied with a novel approach to modeling adsorption isotherms. *Appl. Sci.* **10**(5), 1637. 10.3390/app10051637 (2020).

[15] Wu, Z., Li, H., Ming, J. & Zhao, G. Optimization of adsorption of tea polyphenols into oat β-glucan using response surface methodology. *J. Agricult. Food Chem.* **59**(1), 378–385. 10.1021/jf103003q (2011). 10.1021/jf103003q 21126008

[16] Dhanya, R. Quercetin for managing type 2 diabetes and its complications, an insight into multitarget therapy. *Biomed. Pharmacother.* **146**, 112560. 10.1016/j.biopha.2021.112560 (2022). 34953390 10.1016/j.biopha.2021.112560

[17] Upadhyay, T. K. *et al.* In vitro elucidation of antioxidant, antiproliferative, and apoptotic potential of yeast-derived β-1, 3-glucan particles against cervical cancer cells. *Front. Oncol.* **12**, 942075. 10.3389/fonc.2022.942075 (2022). 36059639 10.3389/fonc.2022.942075 PMC9436396

[18] Rahar, S., Swami, G., Nagpal, N. M. A. & Singh, G. S. Preparation, characterization, and biological properties of β-glucans. *J. Adv. Pharm. Technol. Res.* **2**, 94. 10.4103/2231-4040.82953 (2011). 22171300 10.4103/2231-4040.82953 PMC3217690

[19] N. Guizani, M. S. Rahman, M. Klibi, A. Al-Rawahi, S. Bornaz, Thermal characteristics of Agaricus bisporus mushroom: freezing point, glass transition, and maximal-freeze-concentration condition, *Int. Food Res. J.* 20 (2013).

[20] Upadhyay, T. K., Fatima, N., Sharma, D., Saravanakumar, V. & Sharma, R. Preparation and characterization of beta-glucan particles containing a payload of nanoembedded rifabutin for enhanced targeted delivery to macrophages. *EXCLI J.* **16**, 210 (2017). 28507467 10.17179/excli2016-804 PMC5427468

[21] Bhosale, A. *et al.* Investigation on Antimicrobial, Antioxidant, and Anti-cancerous activity of Agaricus bisporus derived β-Glucan particles against cervical cancer cell line. *Cell. Mol. Biol.* **68**, 150–159 (2022). 36905259 10.14715/cmb/2022.68.9.24

[22] Gründemann, C. *et al.* Comparative chemical and biological investigations of β-glucan-containing products from shiitake mushrooms. *J. Funct. Foods.* **18**, 692–702. 10.1016/j.jff.2015.08.022 (2015).

[23] Chen, X., Siu, K. C., Cheung, Y. C. & Wu, J. Y. Structure and properties of a (1→ 3)-β-d-glucan from ultrasound-degraded exopolysaccharides of a medicinal fungus. *Carbohydr. Polym.* **106**, 270–275. 10.1016/j.carbpol.2014.02.040 (2014). 24721078 10.1016/j.carbpol.2014.02.040

[24] Dalvi, A., Ravi, P. R. & Uppuluri, C. T. Design and evaluation of rufinamide nanocrystals loaded thermoresponsive nasal in situ gelling system for improved drug distribution to brain. *Front. Pharmacol.* **13**, 943772. 10.3389/fphar.2022.943772 (2022). 36267292 10.3389/fphar.2022.943772 PMC9577085

[25] Mahmoud Amer, E. *et al.* Enhancement of β-glucan biological activity using a modified acid-base extraction method from *Saccharomyces cerevisiae*. *Molecules.* **26**, 2113. 10.3390/molecules26082113 (2021). 33917024 10.3390/molecules26082113 PMC8067753

[26] Kaplan-Ashiri, I. SEM-EDS mapping at the nanoscale–the low voltage approach. *Microsc. Microanal.* **28**, 570–571. 10.1017/s1431927622002859 (2022).

[27] Anusuya, S. & Sathiyabama, M. Preparation of β-d-glucan nanoparticles and its antifungal activity. *Int. J. Biol. Macromol.* **70**, 440–443. 10.1016/j.ijbiomac.2014.07.011 (2014). 25036603 10.1016/j.ijbiomac.2014.07.011

[28] Pérez-Bassart, Z., Fabra, M. J., Martínez-Abad, A. & López-Rubio, A. Compositional differences of β-glucan-rich extracts from three relevant mushrooms obtained through a sequential extraction protocol. *Food Chem.* **402**, 134207. 10.1016/j.foodchem.2022.134207 (2023). 36126575 10.1016/j.foodchem.2022.134207

[29] Zhao, L., Lin, S., Lin, J., Wu, J. & Chen, H. Effect of acid hydrolysis on the structural and antioxidant characteristics of β-glucan extracted from Qingke (*Tibetan hulless* barley). *Front. Nutr.* **9**, 1052901. 10.3389/fnut.2022.1052901 (2022). 36438764 10.3389/fnut.2022.1052901 PMC9691401

[30] Raj, V. & Prabha, G. Synthesis, characterization and in vitro drug release of cisplatin loaded Cassava starch acetate–PEG/gelatin nanocomposites. *J. Assoc. Arab Univ. Basic Appl. Sci.* **21**, 10–16. 10.1016/j.jaubas.2015.08.001 (2016).

[31] Sereena, M. C. & Sebastian, D. Evaluation of anticancer and anti-hemolytic activity of azurin, a novel bacterial protein from *Pseudomonas aeruginosa* SSj. *Int. J. Pept. Res. Ther.* **26**, 459–466. 10.1007/s10989-019-09851-1 (2020).

[32] Khan, A. A. *et al.* Effect of γ-irradiation on structural, functional and antioxidant properties of β-glucan extracted from button mushroom (*Agaricus bisporus*). *Innov. Food Sci. Emerg. Technol.* **31**, 123–130. 10.1016/j.ifset.2015.05.006 (2015).

[33] Lin, S. *et al.* Phenolic profiles, β-glucan contents, and antioxidant capacities of colored Qingke (Tibetan hulless barley) cultivars. *J. Cereal Sci.* **81**, 69–75. 10.1016/j.jcs.2018.04.001 (2018).

[34] Atmaca, G. Y., Aksel, M., Bilgin, M. D. & Erdoğmuş, A. Comparison of sonodynamic, photodynamic and sonophotodynamic therapy activity of fluorinated pyridine substituted silicon phthalocyanines on PC3 prostate cancer cell line. *Photodiagnosis Photodyn Ther.* **42**, 103339. 10.1016/j.pdpdt.2023.103339 (2023). 36781009 10.1016/j.pdpdt.2023.103339

[35] Liu, Z., Chen, H., Yang, H., Liang, J. & Li, X. Low-dose UVA radiation-induced adaptive response in cultured human dermal fibroblasts. *Int. J. Photoenergy.*10.1155/2012/167425 (2012).

[36] Zhang, Y. *et al.* Paclitaxel induces the apoptosis of prostate cancer cells via ROS-mediated HIF-1α expression. *Molecules* **27**, 7183. 10.3390/molecules27217183 (2022). 36364008 10.3390/molecules27217183 PMC9654100

[37] Ghaffari, M. *et al.* Co-delivery of curcumin and Bcl-2 siRNA by PAMAM dendrimers for enhancement of the therapeutic efficacy in HeLa cancer cells. *Colloids Surf. B.* **188**, 110762. 10.1016/j.colsurfb.2019.110762 (2020). 10.1016/j.colsurfb.2019.110762 PMC7071519 31911391

[38] Yang, Q. *et al.* Exposure to zinc induces lysosomal-mitochondrial axis-mediated apoptosis in PK-15 cells. *Ecotoxicol. Environ. Saf.* **241**, 113716. 10.1016/j.ecoenv.2022.113716 (2022). 35667309 10.1016/j.ecoenv.2022.113716

[39] Baker, A., Khalid, M., Uddin, I. & Khan, M. S. Targeted non-AR mediated smart delivery of abiraterone to the prostate cancer. *Plos one* **17**, e0272396. 10.1371/journal.pone.0272396 (2022). 36018864 10.1371/journal.pone.0272396 PMC9416994

[40] Pitchai, D., Roy, A. & Ignatius, C. In vitro evaluation of anticancer potentials of lupeol isolated from Elephantopus scaber L on MCF-7 cell line. *J. Adv. Pharm. Technol. Res.* **5**, 179. 10.4103/2231-4040.143037 (2014). 25364696 10.4103/2231-4040.143037 PMC4215481

[41] Cevik, O., Turut, F. A., Acidereli, H. & Yildirim, S. Cyclosporine-A induces apoptosis in human prostate cancer cells PC3 and DU145 via downregulation of COX-2 and upregulation of TGFβ, Turkish. *J. Biochem.* **44**, 47–54. 10.1515/tjb-2017-0355 (2018).

[42] Bobadilla, A. V. P. *et al.**In vitro* cell migration quantification method for scratch assays. *J. R. Soc. Interface.* **16**, 20180709. 10.1098/rsif.2018.0709 (2019). 30958186 10.1098/rsif.2018.0709 PMC6408363

[43] Saadat, Y. R., Saeidi, N., Vahed, S. Z., Barzegari, A. & Barar, J. An update to DNA ladder assay for apoptosis detection. *BioImpacts: BI.* **5**, 25 (2015). 25901294 10.15171/bi.2015.01 PMC4401164

[44] Boonthatui, Y., Thakhiew, W. & Kittisakulnam, S. Quantitative changes of phenolics flavonoids, β-glucans and antioxidant activities during the first and second harvests of split-gill mushroom (Schizophyllum commune). *Agric. Nat. Resour.* **55**, 995–1004 (2021).

[45] Sun, W. *et al.* The effect of particle size on the absorption of cyclosporin a nanosuspensions. *Int. J. Nanomed.*10.2147/ijn.s357541 (2022). 10.2147/IJN.S357541 PMC9034871 35469173

[46] Liu, Q., Han, C., Tian, Y. & Liu, T. Fabrication of curcumin-loaded zein nanoparticles stabilized by sodium caseinate/sodium alginate: Curcumin solubility, thermal properties, rheology, and stability. *Process Biochem.* **94**, 30–38. 10.1016/j.procbio.2020.03.017 (2020).

[47] Boutros, J. A., Magee, A. S. & Cox, D. Comparison of structural differences between yeast β-glucan sourced from different strains of *Saccharomyces cerevisiae* and processed using proprietary manufacturing processes. *Food Chem.* **367**, 130708. 10.1016/j.foodchem.2021.130708 (2022). 34352692 10.1016/j.foodchem.2021.130708

[48] Jakubowska, E., Gierszewska, M., Szydłowska-Czerniak, A., Nowaczyk, J. & Olewnik-Kruszkowska, E. Development and characterization of active packaging films based on chitosan, plasticizer, and quercetin for repassed oil storage. *Food Chem.* **399**, 133934. 10.1016/j.foodchem.2022.133934 (2023). 35998489 10.1016/j.foodchem.2022.133934

[49] Pan, G., Zhang, G., Shi, Q. & Chen, W. The effect of sodium alginate on chlorite and serpentine in chalcopyrite flotation. *Minerals.* **9**, 196. 10.3390/min9030196 (2019).

[50] Ye, H. & Shen, Z. Membrane wrapping efficiency of elastic nanoparticles during endocytosis: Size and shape matter. *ACS Nano.* **13**, 215–228. 10.1021/acsnano.8b05340 (2019). 30557506 10.1021/acsnano.8b05340

[51] Molole, G. J., Gure, A. & Abdissa, N. Determination of total phenolic content and antioxidant activity of Commiphora mollis (Oliv.) Engl. resin. *BMC Chem.* **16**(1), 48. 10.1186/s13065-022-00841-x (2022). 35752844 10.1186/s13065-022-00841-x PMC9233799

[52] Kuo, C. L. *et al.* Mitochondrial oxidative stress in the tumor microenvironment and cancer immunoescape: Foe or friend?. *J. Biomed. Sci.* **29**(1), 74. 10.1186/s12929-022-00859-2 (2022). 36154922 10.1186/s12929-022-00859-2 PMC9511749

[53] Kari, S. *et al.* Programmed cell death detection methods: A systematic review and a categorical comparison. *Apoptosis* **27**(7), 482–508. 10.1007/s10495-022-01735-y (2022). 35713779 10.1007/s10495-022-01735-y PMC9308588

[54] Zhitomirsky, B., Farber, H. & Assaraf, Y. G. LysoTracker and MitoTracker Red are transport substrates of P-glycoprotein: Implications for anticancer drug design evading multidrug resistance. *J. Cell. Mol. Med.* **22**(4), 2131–2141. 10.1111/jcmm.13485 (2018). 29377455 10.1111/jcmm.13485 PMC5867146

[55] Distelmaier, F. *et al.* Life cell quantification of mitochondrial membrane potential at the single organelle level. *Cytometry Part A: J. Int. Soc. Anal. Cytol.* **73**(2), 129–138. 10.1002/cyto.a.20503 (2008). 10.1002/cyto.a.20503 18163486

[56] Zhivotosky, B. & Orrenius, S. Assessment of apoptosis and necrosis by DNA fragmentation and morphological criteria. *Curr. Protoc. Cell Biol.* **12**, 18.3.1-18.3.23. 10.1002/0471143030.cb1803s12 (2001). 10.1002/0471143030.cb1803s12 18228342

[57] Kanagawa, M. *et al.* Structural insights into recognition of triple-helical β-glucans by an insect fungal receptor. *J. Biol. Chem.* **286**(33), 29158–29165. 10.1074/jbc.m111.256701 (2011). 21697086 10.1074/jbc.M111.256701 PMC3190723

[58] Morales, D. *et al.* Isolation and comparison of α-and β-D-glucans from shiitake mushrooms (*Lentinula edodes*) with different biological activities. *Carbohydr. Polym.* **229**, 115521. 10.1016/j.carbpol.2019.115521 (2020). 31826486 10.1016/j.carbpol.2019.115521

[59] Fissan, H., Ristig, S., Kaminski, H., Asbach, C. & Epple, M. Comparison of different characterization methods for nanoparticle dispersions before and after aerosolization. *Anal. Methods* **6**(18), 7324–7334. 10.1039/c4ay01203h (2014). 40883942 10.1039/c4ay01203h

[60] Zhang, S. & Wang, C. Effect of stirring speed on particle dispersion in silica synthesis. *Nano-Struct. Nano-Objects.* **35**, 100994. 10.1016/j.nanoso.2023.100994 (2023).

[61] Paranthaman, S. *et al.* Anti-proliferative potential of Quercetin loaded polymeric mixed micelles on rat C6 and human U87MG glioma cells. *Pharmaceutics.* **14**, 1643. 10.3390/pharmaceutics14081643 (2022). 36015268 10.3390/pharmaceutics14081643 PMC9412540

[62] Almalik, A., Alradwan, I., Kalam, M. A. & Alshamsan, A. Effect of cryoprotection on particle size stability and preservation of chitosan nanoparticles with and without hyaluronate or alginate coating. *Saudi Pharm. J.* **25**, 861–867. 10.1016/j.jsps.2016.12.008 (2017). 28951671 10.1016/j.jsps.2016.12.008 PMC5605945

[63] Tomou, E. M. *et al.* Recent advances in nanoformulations for quercetin delivery. *Pharmaceutics* **15**(6), 1656. 10.3390/pharmaceutics15061656 (2023). 37376104 10.3390/pharmaceutics15061656 PMC10302355

[64] Dian, L. *et al.* Enhancing oral bioavailability of quercetin using novel soluplus polymeric micelles. *Nanoscale Res. Lett.* **9**, 1–11. 10.1186/1556-276x-9-684 (2014). 26088982 10.1186/1556-276X-9-684 PMC4493852

[65] Son, H. Y. *et al.* Formulation and characterization of quercetin-loaded oil in water nanoemulsion and evaluation of hypocholesterolemic activity in rats. *Nutrients* **11**(2), 244. 10.3390/nu11020244 (2019). 30678282 10.3390/nu11020244 PMC6412563

[66] Yan, M., Chen, T., Zhang, S., Lu, T. & Sun, X. A core-shell structured alginate hydrogel beads with tunable thickness of carboxymethyl cellulose coating for pH responsive drug delivery. *J. Biomater. Sci. Polym. Ed.* **32**, 763–778. 10.1080/09205063.2020.1866350 (2021). 33345720 10.1080/09205063.2020.1866350

[67] Kopiasz, Ł *et al.* Effects of dietary oat beta-glucans on colon apoptosis and autophagy through TLRs and dectin-1 signaling pathways—crohn’s disease model study. *Nutrients.* **13**, 321. 10.3390/nu13020321 (2021). 33499397 10.3390/nu13020321 PMC7911679

[68] Kruk, K. & Winnicka, K. Alginates combined with natural polymers as valuable drug delivery platforms. *Mar. Drugs.* **21**, 11. 10.3390/md21010011 (2022). 36662184 10.3390/md21010011 PMC9861938

[69] Azeem, M. *et al.* An insight into anticancer, antioxidant, antimicrobial, antidiabetic and anti-inflammatory effects of quercetin: A review. *Polym. Bull.* **80**, 241–262. 10.1007/s00289-022-04091-8 (2023). 10.1007/s00289-022-04091-8 PMC8800825 35125574

[70] Biswas, P. *et al.* A comprehensive analysis and anti-cancer activities of quercetin in ROS-mediated cancer and cancer stem cells. *Int. J. Mol. Sci.* **23**, 11746. 10.3390/ijms231911746 (2022). 36233051 10.3390/ijms231911746 PMC9569933

[71] Teekaraman, D., Elayapillai, S. P., Viswanathan, M. P. & Jagadeesan, A. Quercetin inhibits human metastatic ovarian cancer cell growth and modulates components of the intrinsic apoptotic pathway in PA-1 cell line. *Chem. Biol. Interact.* **300**, 91–100. 10.1016/j.cbi.2019.01.008 (2019). 30639267 10.1016/j.cbi.2019.01.008

[72] Yadav, S., Sadique, M. A., Pal, M., Khan, R. & Srivastava, A. K. Cytotoxicity and DNA fragmentation-mediated apoptosis response of hexagonal ZnO nanorods against human prostate cancer cells. *Appl. Surf. Sci.* **9**, 100237. 10.1016/j.apsadv.2022.100237 (2022).

