# Author Correction: Preparation, characterization and antioxidant and anticancerous potential of Quercetin loaded β-glucan particles derived from mushroom and yeast

**DOI:** 10.1038/s41598-025-21295-w

**Published:** 2025-10-06

**Authors:** Rashmi Trivedi, Tarun Kumar Upadhyay

**Affiliations:** Department of Biotechnology, Parul Institute of Applied Sciences and Research and Development Cell, Parul University, Vadodara, Gujarat 391760 India

Correction to: *Scientific Reports* 10.1038/s41598-024-66824-1, published online 11 July 2024

The original version of this Article contained an error in Figure 20b.

As a result of an error during figure assembly, the panel for 62.5pg/ml Y3 Control was a duplication of the image for the 125pg/ml Quercetin panel. Figure 20 is now updated.

The original Figure 20 and accompanying legend appear below.

**Fig. 20:**
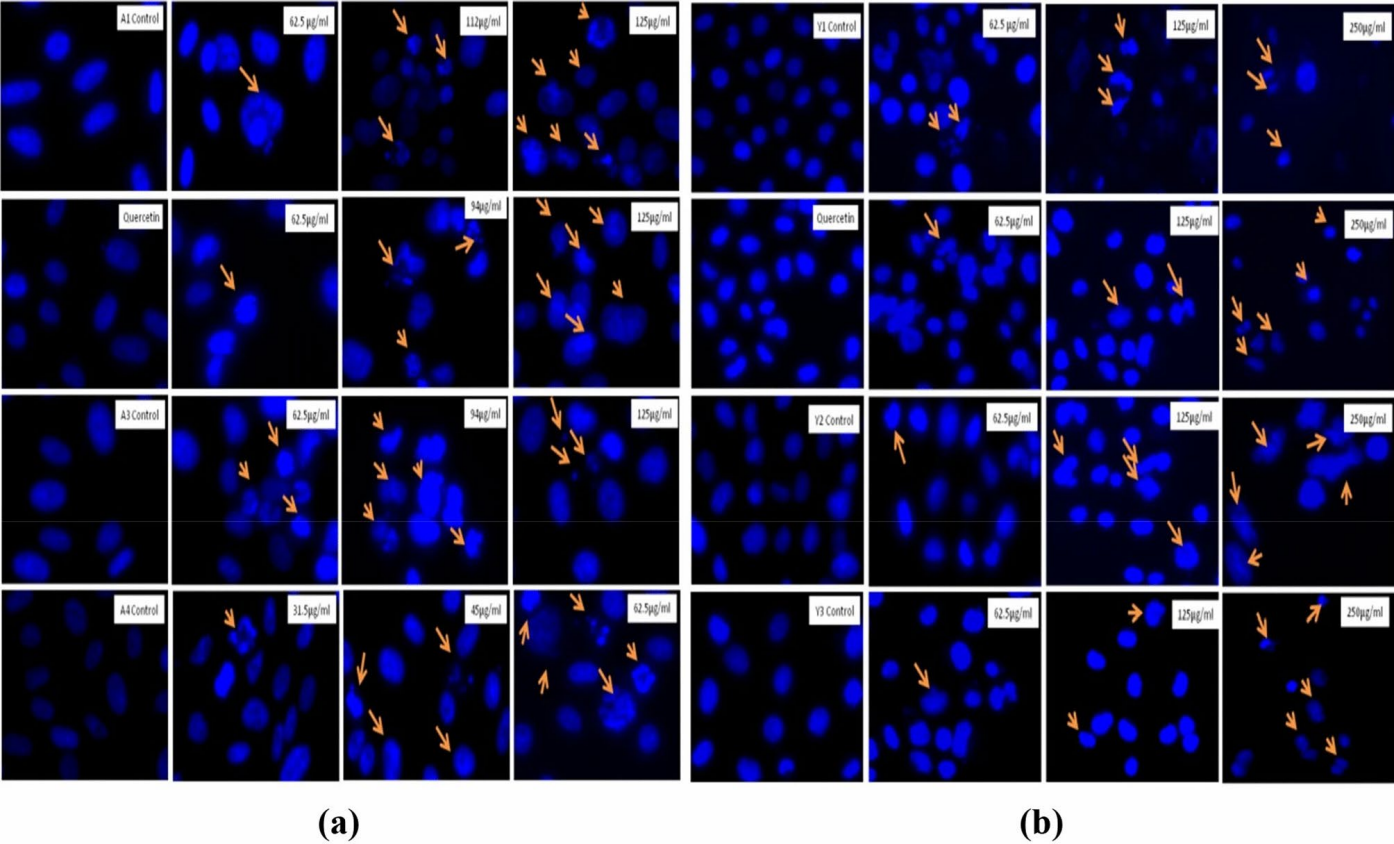
Nuclear morphology changes and DNA fragmentation in the PC3 cells as observed with the microscope. (**a**) PC3 cells after treatment with Agaricus bisporus derived particles. (**b**) PC3 cells after treatment with yeast derived particles. Condensation of the nuclear material and DNA fragmentation is shown with the help of arrows.

The original Article has been corrected.

