# Supplementary material for: Preparation, characterization and antioxidant and anticancerous potential of Quercetin loaded β-glucan particles derived from mushroom and yeast
Source: Sci Rep. 2024 Jul 11;14:16047. doi: 10.1038/s41598-024-66824-1 (PMC11239821; doi:10.1038/s41598-024-66824-1)
Supplement: Supplementary file 1 — Supplementary Information 1. [file 41598_2024_66824_MOESM1_ESM.docx]

(a)


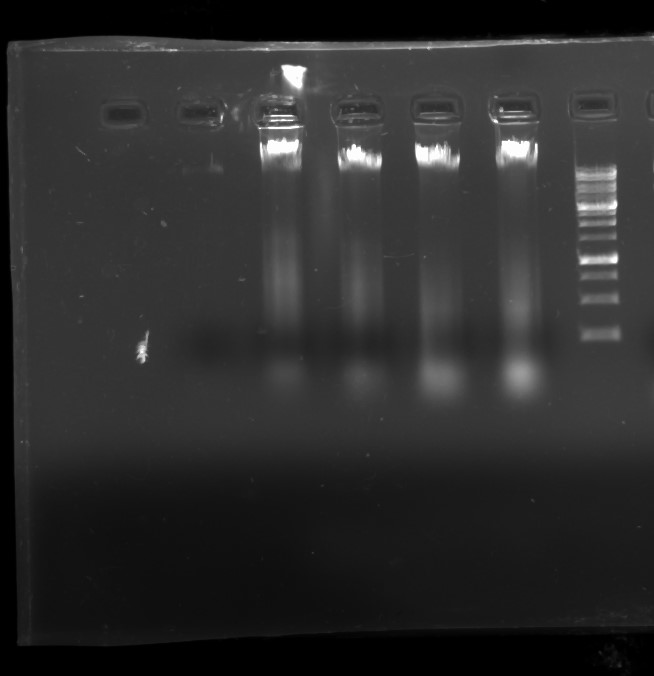


6

5

4

3

2

1

(b)

**Supplementary file Figure 26:** Original gel image of the DNA fragmentation. Lane 1 is control, lane 2-A2, lane 3-A3, lane 4-Y2, lane 5-Y3, lane 5-DNA ladder. Rest two lanes are RNA and not related to our experiment. (a) is original unprocessed image (b) cropped image
