# Supplementary material for: Preparation, characterization and antioxidant and anticancerous potential of Quercetin loaded β-glucan particles derived from mushroom and yeast
Source: Sci Rep. 2024 Jul 11;14:16047. doi: 10.1038/s41598-024-66824-1 (PMC11239821; doi:10.1038/s41598-024-66824-1)
Supplement: Supplementary file 2 — Supplementary Information 2. [file 41598_2024_66824_MOESM2_ESM.docx]

**(A) (B) (C) (D)**

**(E) (F) (G) (H)**

**Supplementary Figure 1: Statistical analysis of Reactive oxygen species (ROS) generation**

(A, B, C, D) are the statistical analysis of the A1, Quercetin, A2, and A3. (E, F, G, H) are the statistical analysis of the Y1, Quercetin, Y2, and Y3. All the values were highly significant with p value less than 0.0005 (*p<0.0005*)

(A) (B) (C) (D)

(E) (F) (G) (H)

**Supplementary Figure 2. Statistical Analysis of Apoptosis detection with Propidium iodide (PI) staining**

(A, B, C, D) are the statistical analysis of the A1, Quercetin, A2, and A3 (E, F, G, H) are the statistical analysis of the Y1, Quercetin, Y2, and Y3. All the values were highly significant with p value less than 0.05 (*p<0.05*)

(A) (B) (C) (D)

(E) (F) (G) (H)

**Supplementary Figure 3: Statistical Analysis of Acidic organelles activity analysis through LysoTracker Red DND-99**

(A, B, C, D) are the statistical analysis of the A1, Quercetin, A2, and A3. (E, F, G, H) are the statistical analysis of the Y1, Quercetin, Y2, and Y3. (A) and (B) were highly significant with p value less than 0.05 (*p<0.05*) while C was non-significant with (*p>0.05*). (D) was found to be less significant. (E) and (F) were less significant while (G) and (H) were highly significant.

(A) (B) (C) (D)

(E) (F) (G) (H)

**Supplementary Figure 4: Statistical analysis of Altered Mitochondrial membrane potential observed through MitoTracker Red CMX-ROS**

(A, B, C, D) are the statistical analysis of the A1, Quercetin, A2, and A3. (E, F, G, H) are the statistical analysis of the Y1, Quercetin, Y2, and Y3. (A) and (B) were less significant with p value less than 0.05 (*p<0.05*) while C was least significant with (*p<0.05*). (D) was found to be highly significant. (E) and (H) were highly significant while (F) and (G) were less significant.
